# A robust and accurate surrogate method for monitoring the frequency and duration of combined sewer overflows

**DOI:** 10.1007/s10661-018-6589-3

**Published:** 2018-03-11

**Authors:** Thomas Hofer, Albert Montserrat, Guenter Gruber, Valentin Gamerith, Lluis Corominas, Dirk Muschalla

**Affiliations:** 1 Institute of Urban Water Management and Landscape Water Engineering, Graz University of Technology, Stremayrgasse 10/I, 8010 Graz, Austria; 2 Catalan Institute for Water Research, Scientific and Technological Park of the University of Girona, H2O Building, Emili Grahit 101, 17003 Girona, Spain; 3 Hydroconsult GmbH, Engineering Company for Environmental Engineering and Water Management, St. Veiter Straße 11a, 8045 Graz, Austria

**Keywords:** Combined sewer overflow, Event detection, Low-cost, Monitoring, Temperature, Surrogate measurement

## Abstract

Discharges of untreated wastewater from combined sewer overflows (CSOs) can affect hydraulic stress and have significant environmental impacts on receiving water bodies. Common flow rate and water level sensors for monitoring of CSO events are expensive in terms of investment costs, installation, operation and maintenance. This paper presents a novel surrogate method to detect CSO events by using two low-cost temperature sensors. The novelty is the experimental setup for installation of temperature sensors in CSO structures and an algorithm developed to automatically calculate the duration of CSO events considering the response time of the system. The occurrence and duration of CSO events is computed based on the convergence of the two temperature signals. The method was tested under field conditions in a CSO structure, and the results were compared to the information gathered from a parallel installed flow sensor. The application of two temperature sensors installed inside a CSO structure was proven to be robust and accurate for the automatic detection of the occurrence and duration of CSO events. Within the 7-month test phase, 100% of the 20 CSO events could be detected without false detections. The accuracy of detecting the start and end of the CSO events was 2 min in comparison to the flow sensor.

**Electronic supplementary material:**

The online version of this article (10.1007/s10661-018-6589-3) contains supplementary material, which is available to authorized users.

## Introduction

Discharges of untreated wastewater from combined sewer overflows (CSOs) can lead to hydraulic stress, oxygen depletion or a temporary increase of pollutant concentrations in receiving waters (Ellis and Hvitved-Jacobsen 1996). The European Urban Waste Water Treatment Directive (UWWTD) 91/271/EEC (EC 1991) indicates that member states will decide on measures to limit pollution from CSOs, which could be based on higher dilution rates, improvement of plant treatment capacity and regulation of the overflow (spill) frequency (Zabel et al. 2001). The directive does not define standards at European level, but since the implementation of the Water Framework Directive (EC 2000), the characterisation of CSO events is of great importance. In countries such as Belgium, Denmark, parts of Germany and the Netherlands, the overflow frequency and partly also overflow duration are used as design criteria for CSO structures (Dirckx et al. 2011).

Online monitoring is the basis to fully understand the behaviour of CSO structures (Gruber et al. 2005; Caradot et al. 2015). The collected information can be used at operational level for function control, quality assessment, prior determination for CSO maintenance and improvement of the sewer system while implementing control strategies (Alferes et al. 2013; Benedetti et al. 2013). The information is also useful to assess, improve and maintain combined sewer systems (e.g. Montserrat et al. 2015) as well as calibrating hydraulic urban drainage models (Duchesne et al. 2001; Montserrat et al. 2017).

Usually flow or water level sensors are widely used for CSO monitoring. These systems allow to detect occurrence, duration, volume and dynamics of a CSO event. Generally, these sensors are expensive in terms of purchase, installation and maintenance, especially in application in urban drainage systems (US-EPA 1999).

Sensors have become increasingly cheaper and more compact in design in recent years (Eggimann et al. 2017). Kinzli et al. (2016) concluded that low-cost water level sensors in sewer systems are available, but show limited robustness. They experienced several practical problems due to mechanical stress, deficiencies in long-term waterproofness, condensation resistance as well as measurement failures due to sensor fouling. In addition, level sensors usually require an available power supply on site, which implies higher investment and operational costs. Potential extra costs may arise due to an explosion proofed sensor design in regions where this is mandatory. Due to these facts the applicability of such low-cost sensors and further the simultaneous CSO detection in urban drainage systems are limited.

Therefore, a variety of publications attended on alternative (surrogate) methods for CSO detection, which can be classified in three categories depending on the place of measurement: (i) CSO detection methods in the sewer system, (ii) CSO detection methods in the receiving water body and (iii) model-based CSO detection methods.

### CSO detection methods in the sewer system

Rasmussen et al. (2008) used two electrical contacts as a simple switch in the CSO structure. The installation can be on the weir crest or in the overflow channel. Investment costs excluding data transmission are approximately 200 € and no preconditions (advance information) are required. Potential false detections by clogging of the electrical contacts can significantly disturb the measurement.

Siemers et al. (2011) used motion sensors to detect flow acceleration in the CSO structure. Sensor costs are about 200 € and also no preconditions are required but potential false detections by wind in the sewer system are possible.

Wani et al. (2017) used moisture sensors for CSO detection. Sensor costs are in a range of 100–300 € and again no preconditions or advance information are required. Potential false detections by a changing air humidity in the sewer system are possible, where a low signal-to-noise ratio can be observed.

Gruber et al. (2005) used water quality sensors for CSO detection. Additional to a CSO detection also pollutant concentrations and dynamics can be observed by this method. The sensor costs are at least 15,000 € and a high effort in operation is needed.

Jeanbourquin et al. (2011) and Lo et al. (2015) used video image analysis for flow detection in the CSO structure. The costs are around 1000 €, a large amount of data as well as a high effort in data evaluation is needed.

Montserrat et al. (2013) proposed an approach of using a single low-cost temperature sensor installed in the CSO structure. The detection of a CSO event is based on the identification of an abrupt change in the temperature signal. The investment costs without data transmission are about 50 € and no advance information is required. Potential false detections by similar air and waste water temperature (low signal-to-noise ratio) are possible.

A brief summary of the described methods is given in Table 1.

**Table 1:** Compilation of methods for CSO detection in the sewer system

Publication	CSO detection criteria	Signal type	CSO occurrence	CSO duration	CSO volume
Rasmussen et al. (2008)	Short-circuit bridge of electrical contacts	Binary (0/1)	Yes	Yes	No
Siemers et al. (2011)	Critical acceleration of sensor	Binary (0/1)	Yes	Yes	No
Wani et al. (2017)	Critical gradient of moisture signal	Binary (0/1)	Yes	Yes	No
Gruber et al. (2005)	Critical pollution concentration (by dillution)	Binary (0/1)	Yes	Yes	No
Jeanbourquin et al. (2011)	Location change of surface particles over time	Binary (0/1)	Yes	Yes	Yes
Lo et al. (2015)	Visual sensing combined with virtual image markers	Binary (0/1)	Yes	Yes	No
Montserrat et al. (2013)	Critical gradient of temperature shift	Binary (0/1)	Yes	Yes	No

### CSO detection methods in the receiving water body

The following methods for CSO detection in the receiving water body give information about the detection of a CSO occurrence. Detections of CSO duration and CSO volume were not evaluated in these studies. Dependent on the chosen detection parameter (e. g. temperature, caffeine concentration or others) a simultaneous measurement of the already existing background exposure of the chosen parameter in the receiving water body is needed to distinguish between periods with and without CSO influence.

Schilperoort et al. (2006) and Riechel et al. (2010) conducted CSO detection by using temperature sensors in the receiving water body. The sensor invest costs are around 500 € but potential false detections by similar river and waste water temperature are possible (low signal-to-noise ratio).

Buerge et al. (2006) and Weyrauch et al. (2010) used information from incremental samples and lab analysis of selected micro pollutants like caffeine. These methods based on incremental samples are not suitable for a continuous CSO monitoring. The costs are approximately 100 € per lab analysis excluding sampling costs.

### Model-based CSO detection methods

CSO events can be detected indirectly by model-based approaches. Due to the complexity of the used models the need of sufficient data and a mandatory model calibration are general constraints for these methods. The advantage of such approaches is the possibility to apply the available model for the comparison of different CSO management scenarios in the system.

Thorndahl and Willems (2008), Schroeder et al. (2011) and Yu et al. (2013) used a model approach of rain hyetographs by rainfall depth and rainfall duration time in combination with a hydrodynamic sewer model for CSO detection. The developed model can furthermore be applied to develop future management strategies. However, rainfall data of sufficient duration and quality and a calibrated hydrodynamic sewer simulation model including CSO structures are needed as a precondition, which potentially demands a high effort in model setup and model calibration. A compilation of the described methods is given in Table 2.

**Table 2:** Compilation of model based methods for CSO detection

Publication	CSO detection criteria	Signal type	CSO occurrence	CSO duration	CSO volume
Thorndahl and Willems (2008)	Critical rainfall height and rainfall duration time	Binary (0/1)	Yes	Yes	No
Schroeder et al. (2011)	Critical rainfall height	Binary (0/1)	Yes	Yes	No
Yu et al. (2013)	Critical accordance of rainfall data and CSO data	Analog/digital	Yes	Yes	Yes

Montserrat et al. (2013) presented the application of a single low-cost temperature sensor installed inside a CSO structure to detect CSO events for the first time. That approach bases the detection of a CSO event on the identification of an abrupt change in the temperature signal. Although results were reasonable for most of the evaluated CSO structures, the approach led to false detections when the signal-to-noise ratio was low.

This paper increases the robustness of the method proposed in Montserrat et al. (2013) by adding a second temperature sensor, improves the detection accuracy by implementing an algorithm that accounts for the response time of the system and automatically calculates the duration of CSO events.

## Materials and methods

The developed method requires the simultaneous measurement signals of two temperature sensors in a CSO structure. The first temperature sensor (*S*_1_) is submerged into the wastewater stream to continuously measure the wastewater temperature. The second temperature sensor (*S*_2_) is installed on the crest of the overflow weir or on the invert of the CSO overflow channel to alternatively measure the temperature of ambient air during dry weather conditions (sensor *S*_2_ is not submerged) and the temperature of the overflowing wastewater during a CSO event (sensor S_2_ is submerged).

In this paper, a CSO event is defined as a discharge process from the drainage system to the receiving water body. This implicates that one or more CSO events may occur during one rainfall event.

The stated methodology is based on two assumptions:

(i) Sensor behaviour in dry weather conditions: Sensor *S*_1_ measures the wastewater temperature *T*_S1_, and sensor *S*_2_ measures the ambient air temperature *T*_S2_. The temperature signals of sensors *S*_1_ and *S*_2_ differ in dry weather conditions.
(ii) Sensor behaviour during a CSO event: Both sensors *S*_1_ and *S*_2_ measure the temperature of the wastewater. The temperature signals of sensors *S*_1_ and *S*_2_ converge during a CSO event within a defined temperature difference.

### Description of the algorithm for data analysis

An algorithm was developed based on the stated method to treat the gathered data from the temperature sensors and calculate the occurrence and duration of CSO events. The algorithm detects the convergence of the temperature signals *T*_S1_ and *T*_S2_ during a CSO event. In addition, the algorithm includes heat transfer equations to minimize the time delay in the detection.

**Fig. 1:**
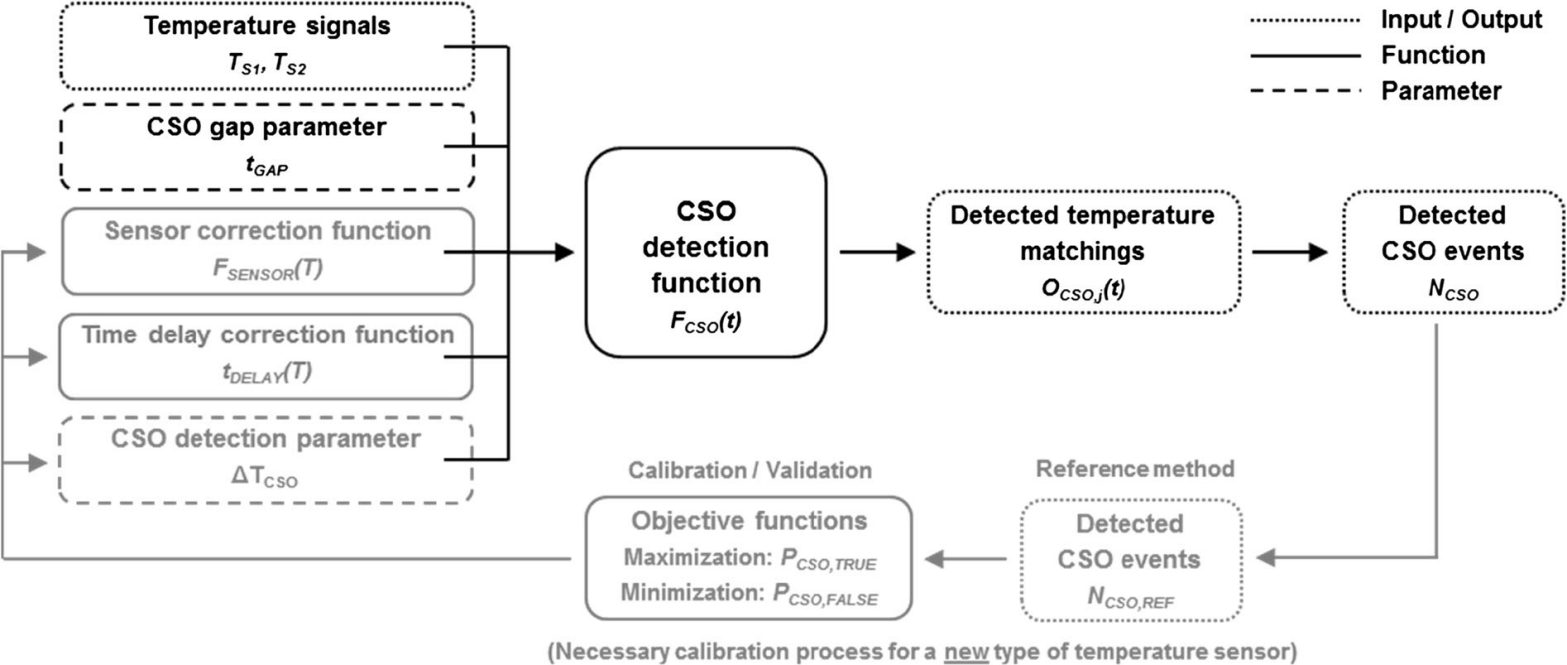
Conceptual scheme of the algorithm for CSO detection

Figure 1 shows the conceptual scheme of the algorithm. The three different types of elements in the algorithm are inputs and outputs (dotted frame), functions (continuous frame) and parameters (dashed frame). The grey fields show the elements necessary to calibrate the algorithm if a new type of temperature sensor is used.

#### Sensor correction function *F*_SENSOR_(*T*)

The sensor correction function *F*_SENSOR_(*T*) considers the potential deviations of the measured temperature signals *T*_S1_ and *T*_S2_, which depends on the accuracy of the specific sensor type. The determination of this function has to be done only once for a new type of temperature sensor.

Since this function depends on the current temperature, measurements have to be performed over a range of different temperature levels *j* (see Eq. 1).

1 $$ {F}_{\mathrm{S}\mathrm{ENSOR}}(T)={\left[{T}_{\mathrm{S}1}\left({T}_j\right)-{T}_{\mathrm{S}2}\left({T}_j\right)\right]}_{j=1}^{j=n} $$

with *F*_SENSOR_(*T*): sensor correction function; *T*_S1_(*T*_*j*_): temperature signal of sensor *S*_1_ for temperature *T*_*j*_; *T*_S2_(*T*_*j*_): temperature signal of sensor *S*_2_ for temperature *T*_*j*_; *j*: temperature level.

#### Time delay correction function *t*_DELAY_(*T*)

The temperature convergence of a body in an environment with constant temperature *T*_ENV_ can be determined by Newton’s law of cooling (O’Connell and Haile 2005). This effect becomes relevant during the start and end of a CSO event. Sensor *S*_2_ is situated on the crest of the overflow weir or on the invert of the CSO overflow channel. Therefore, as a CSO event initiates, the temperature changes abruptly from the air temperature to the overflowing wastewater temperature, and the opposite happens at the end of a CSO event.

The temperature convergence *T*(*t*) of two temperature signals can be described by Eq. 2.

2 $$ T(t)={T}_{\mathrm{ENV}}+\left(T(0)-{T}_{\mathrm{ENV}}\right)\ast {e}^{-\frac{t}{\tau }}\kern3.25em with\ \tau =\frac{\rho \ast {c}_p\ast V}{h\ast A} $$

with *T*(*t*): temperature of the body at time *t*; *T*_ENV_: temperature of the environment; *T*(0): initial temperature of the body with converging temperature at time = 0; *τ*: time constant (*t*); *ρ*: density of medium (gm^−3^); *c*_p_: isobaric mass heat capacity (Jg^−1^ K^−1^); *V*: body volume (m^3^); *h*: heat transfer coefficient (Js^−1^m^−2^K^−1^); *A*: heat transfer surface area (m^2^).

The time constant τ describes the physical system characteristics of heat transfer from water to air and from air to water for the specific installation technique and specific temperature sensors used. It is dependent on the density *ρ* of the medium (gm^−3^), the isobaric mass heat capacity *c*_p_ (Jg^−1^ K^−1^), the body volume *V* (m^3^), the heat transfer coefficient *h* (Js^−1^m^−2^K^−1^) and the heat transfer surface area *A* (m^2^).

To describe the duration until convergence of two temperature signals in the same environmental conditions, Eq. 2 can be transformed to Eq. 3, assuming that the temperature of the environment is equal to the measured temperature of sensor *S*_1_ (*T*_ENV_ = *T*_S1_) and the temperature of the body is equal to the measured temperature of sensor *S*_2_ (*T* = *T*_S2_). The duration until convergence is equal to the time delay parameter *t*_DELAY_(*T*).

The determination of the parameters for this function has to be done only one-time for a new type of temperature sensor.

3 $$ {t}_{\mathrm{DELAY}}(T)=\ln \frac{T_{\mathrm{S}2}(0)-{T}_{\mathrm{S}1}}{T_{\mathrm{S}2}(t)-{T}_{\mathrm{S}1}}\ast \tau $$

with *t*_DELAY_(*T*): time delay parameter; *T*_ENV_: temperature of the ambient air; *T*(0): initial temperature of the body with converging temperature at time = 0; *τ*: time constant (*t*).

#### CSO gap parameter *t*_GAP_

The CSO gap parameter *t*_GAP_ defines the maximum time gap between CSO occurrences within which they are summarized as a single CSO event. This parameter is dependent on the system’s dynamics and independent of the used sensor type.

#### CSO detection parameter Δ*T*_CSO_

The CSO detection parameter Δ*T*_CSO_ defines the maximum temperature difference between two temperature signals, *T*_S1_ and *T*_S2_, within which the algorithm will detect the start and end of a CSO event. It is dependent on sensor specifications as accuracy, resolution, stability and time-synchronicity. Once calibrated for a type of sensor, it is independent from the measurement location.

#### CSO detection function *F*_CSO_(*t*)

The CSO detection function *F*_CSO_(*t*) is defined as a binary index vector by the ratio of the temperature difference function ΔT(*t*) and CSO detection parameter Δ*T*_CSO_. The temperature difference function ΔT(*t*) is defined in Eq. 4 by subtracting the temperature signals *T*_S1_ and *T*_S2_. Potential sensor deviations are considered by adding the sensor correction function *F*_SENSOR_(*T*).

4 $$ \varDelta T(t)={T}_{\mathrm{S}1}(t)-{T}_{\mathrm{S}2}(t)+{F}_{\mathrm{S}\mathrm{ENSOR}}\left({T}_{\mathrm{S}1}(t)\right) $$

With Δ*T*(*t*): temperature difference function; *T*_S1_(t): temperature signal of sensor *S*_1_; *T*_S2_(*t*): temperature signal of sensor *S*_2_; *F*_SENSOR_(*t*): sensor correction function.

For time *t*, a ratio less than or equal to 1.0 results in a value of 1, which is tagged as a temperature match event. A ratio higher than 1.0 results in a value of 0, indicating no temperature match. The functional relationship is stated in Eq. 5.

5 $$ {F}_{CSO}(t)=\left\{\begin{array}{c}\ 1,\kern0.5em \frac{\varDelta T(t)}{\varDelta {T}_{CSO}}\le 1\\ {}0,\kern0.5em \frac{\varDelta T(t)}{\varDelta {T}_{CSO}}>1\end{array}\right. $$

with *F*_CSO_(*t*): CSO detection function; Δ*T*(*t*): temperature difference function; Δ*T*_CSO_: CSO detection parameter.

#### Index vector of detected temperature matches *O*_CSO_

The index vector of detected temperature matches during a CSO event *j* is defined as *O*_CSO,j_. Each index *F*_CSO_(*t*_*i*_) = 1 within the defined maximum time gap *t*_GAP_ is counted as a temperature match of a CSO event *j*. The time delay correction is considered by subtracting the time delay parameter *t*_DELAY_(*T*) (see Eq. 6).

6 $$ {O}_{\mathrm{CSO},j}(t)={\left[{F}_{\mathrm{CSO}}{\left({t}_i\right)}_j=1\right]}_{i=1}^{i=n}-{t}_{\mathrm{DELAY}}(T)\kern2.75em with{t}_{i+1}-{t}_i\le {t}_{\mathrm{GAP}} $$

with *O*_CSO,*j*_(*t*): index vector of detected temperature matches during a CSO event *j*; *F*_CSO_(*t*): CSO detection function at time step *i*; *t*_GAP_: CSO gap parameter; *t*_DELAY_(*T*): time delay correction function.

#### Number of detected CSO events *N*_CSO_

The number of detected CSO events N_CSO_ is defined as the sum of detected index vectors *O*_CSO,j_ (see Eq. 7).

7 $$ {N}_{\mathrm{CSO}}=\sum \limits_{j=1}^n{O}_{\mathrm{CSO},j}(t) $$

with *N*_CSO_: number of detected CSO events; *O*_CSO,j_(*t*): index vector of detected temperature matches during a CSO event *j*.

### Calibration and validation process of the algorithm for a new type of temperature sensor

For a new type of temperature sensor, an initial calibration and validation process of the algorithm is required to determine the optimum values for the CSO detection parameter Δ*T*_CSO_ and the CSO gap parameter *t*_GAP_. This is conducted by calibrating against reference data. The calibration process only depends on the type of used temperature sensors and it is independent of the location of detection or the experimental setup.

#### CSO detection function *F*_CSO,REF_(*t*) based on the reference method

The CSO detection function *F*_CSO,REF_(*t*) based on the reference method is defined as a binary index vector, which determines the exceedance of a defined threshold value. The determination of the threshold value is dependent on the selected reference method. For time *t*, a measurement value equal to or higher than the threshold value results in a value of 1, which is tagged as a CSO occurrence. A measurement value lower than the threshold value results in a value of 0, which indicates no CSO occurrence. The functional relationship is stated in Eq. 8.

8 $$ {F}_{\mathrm{CSO},\mathrm{REF}}(t)=\left\{\begin{array}{c}\ 1,\kern0.5em f(t)\ge Threshold\\ {}0,\kern0.5em f(t)< Threshold\end{array}\right. $$

with *F*_CSO,REF_(*t*): CSO detection function by the reference method; *f*(*t*): measurement signal by the reference method.

#### Index vector of the detected CSO occurrences by the reference method

The index vector of detected discrete CSO occurrences by the reference method during a CSO event *j* is defined as *O*_CSO,REF,*j*_. Each index *F*_CSO,REF_(*t*_*i*_) = 1 is counted as a discrete CSO occurrence of a CSO event *j* (see Eq. 9).

9 $$ {O}_{\mathrm{CSO},\mathrm{REF},j}(t)={\left[{F}_{\mathrm{CSO},\mathrm{REF}}\left({t}_i\right)=1\right]}_j $$

with *O*_CSO,REF,*j*_(*t*): sum of detected discrete CSO occurrences during a CSO event j by the reference method; *F*_CSO,REF_(*t*): CSO detection function at time step *i* by the reference method.

#### Number of detected CSO events by the reference method

The number of detected CSO events by the reference method is defined as *N*_CSO,REF_ (see Eq. 10).

10 $$ {N}_{\mathrm{CSO},\mathrm{REF}}=\sum \limits_{j=1}^n{O}_{\mathrm{CSO},\mathrm{REF},j}(t) $$

with *N*_CSO,REF_: number of all detected CSO events by the reference method; *O*_CSO,REF,*j*_(*t*): index vector of detected temperature matches during a CSO event *j* by the reference method.

#### Function of true positive and false positive temperature matches *O*_CSO,TRUE_(*t*) and *O*_CSO,FALSE_(*t*)

A fitting temperature match is defined as a simultaneous detection by the algorithm and the reference method. This value can be classified in a more general way as a “true positive” temperature match regarding to statistical hypothesis testing (Fawcett, 2006). An unfitting temperature match is defined as a detection only by the algorithm and not by the reference method. This identified false observation can be indicated as a “false positive” temperature match.

The number of true positive matches *O*_CSO,TRUE_(*t*), where a discrete CSO occurrence is detected by both the algorithm and the reference method is determined by the subtraction of the index vectors *O*_CSO_(*t*) and *O*_CSO,REF_(*t*) and addition of an index vector of value 1. All time steps with a pair of zero values (*O*_CSO_(t) = 0 and *O*_CSO,REF_(t) = 0) have to be removed before the resulting index vectors can be used in the algorithm, because they represent instances of no temperature matches of both methods. The resulting indices of value 1 indicate the true positive temperature matches *O*_CSO,TRUE_(*t*), whereas the indices of value 2 indicate the false positive temperature matches *O*_CSO,FALSE_(*t*) (see Eq. 11).

11 $$ \overrightarrow{O_{\mathrm{CSO}}(t)}-\overrightarrow{O_{\mathrm{CSO},\mathrm{REF}}(t)}+\overrightarrow{1}=\left\{\begin{array}{c}\ 1,\kern0.5em \to {O}_{\mathrm{CSO},\mathrm{TRUE}}(t)\\ {}2,\kern0.5em \to {O}_{\mathrm{CSO},\mathrm{FALSE}}(t)\end{array}\right. $$

with O_CSO_(*t*): index vector of detected temperature matches; *O*_CSO,REF_(*t*): index vector of detected temperature matches by the reference method; *O*_CSO,TRUE_(*t*): index vector of true positive temperature matches; *O*_CSO,FALSE_(*t*): index vector of false positive temperature matches.

#### Objective function 1: percentage of detected CSO events *P*_CSO,EVENTS_ (%)

The percentage of detected CSO events *P*_CSO,EVENTS_ is defined as the percentage of all detected CSO events with respect to the reference method (see Eq. 12). A percentage of 100% indicates a perfect fit of all detected CSO events by both methods.

12 $$ {P}_{\mathrm{CSO},\mathrm{EVENTS}}=\frac{\sum {N}_{\mathrm{CSO}}}{\sum {N}_{\mathrm{CSO},\mathrm{REF}}}\ast 100 $$

with *P*_CSO,EVENTS_: percentage of detected CSO events; *N*_CSO_: number of all detected CSO events; *N*_CSO,REF_: number of all detected CSO events by the reference method.

#### Objective function 2: percentage of true positive matches *P*_CSO,TRUE_ (%)

The percentage of true positive matches *P*_CSO,TRUE_ is defined as the percentage of detected temperature matches in accordance with the reference method (see Eq. 13). A percentage of 100% represents the perfect fit of all detected temperature matches by both methods.

13 $$ {P}_{\mathrm{CSO},\mathrm{TRUE}}=100-\frac{\sum {O}_{\mathrm{CSO},\mathrm{REF}}-\sum {O}_{\mathrm{CSO},\mathrm{TRUE}}}{\sum {O}_{\mathrm{CSO},\mathrm{REF}}}\ast 100\kern0.5em $$

with *P*_CSO,TRUE_: percentage of true positive matches (%); *O*_CSO,REF_: number of detected discrete CSO occurrences by reference method; *O*_CSO,TRUE_: number of true positive matches.

#### Objective function 3: percentage of false positive matches *P*_CSO,FALSE_ (%)

The percentage of false positive matches *P*_CSO,FALSE_ is defined as the percentage of non-compliant detected temperature matches with respect to the reference method (see Eq. 14). A percentage of 0% indicates no false detected temperature matches by the algorithm.

14 $$ {P}_{\mathrm{CSO},\mathrm{FALSE}}=100-\frac{\sum {O}_{\mathrm{CSO},\mathrm{REF}}-\sum {O}_{\mathrm{CSO},\mathrm{FALSE}}}{\sum {O}_{\mathrm{CSO},\mathrm{REF}}}\ast 100\kern0.5em $$

with *P*_CSO,FALSE_: percentage of false positive matches (%); *O*_CSO,REF_: number of detected temperature matches by reference method; *O*_CSO,FALSE_: number of false positive matches.

The performance of the algorithm calibration can be evaluated in a validation process for a sufficient number of CSO events. The same reference method for CSO detection as in the calibration process can be used in parallel to the CSO detection algorithm. To quantify the quality of the validation process, the same three quality criteria as in the calibration process are used.

### Materials

To select the appropriate type of temperature sensor for application in a CSO structure, several attributes should be considered during the selection process. In addition to the investment and operational costs, the sensor size, its robustness and water resistance, effort required for installation, maintenance and data readout, and possibility of an independent power supply and explosion proofed design (if required) are of importance.

For temperature monitoring in this study, two low-cost temperature sensors with an integrated data logger were used in combination with a compatible contactless data reader. The HOBO® Pendant UA-001-64 sensor (Onset Computer Corporation) embedded in a waterproof polypropylene casing (dimensions: 58 × 33 × 23 mm, weight: 18 g) was used as the temperature sensor. The compact dimensions of the sensor should prevent clogging in the sewer system. An independent power supply was provided from a 3 V CR-2032 lithium battery with a durability of about 1 year. The internal memory of 64 kb for logging 52,000 10-bit readings allows continuous data storage for 36 days using a 1-min logging interval (which was chosen as the logging interval for this study). According to the manufacturer, the measurement limits range from – 20 to 50 °C, with an accuracy of ±0.53 °C (drift less than 0.1 °C/year) and a resolution of 0.1 °C.

### Experimental setup — case study Graz (Austria)

**Fig. 2:**
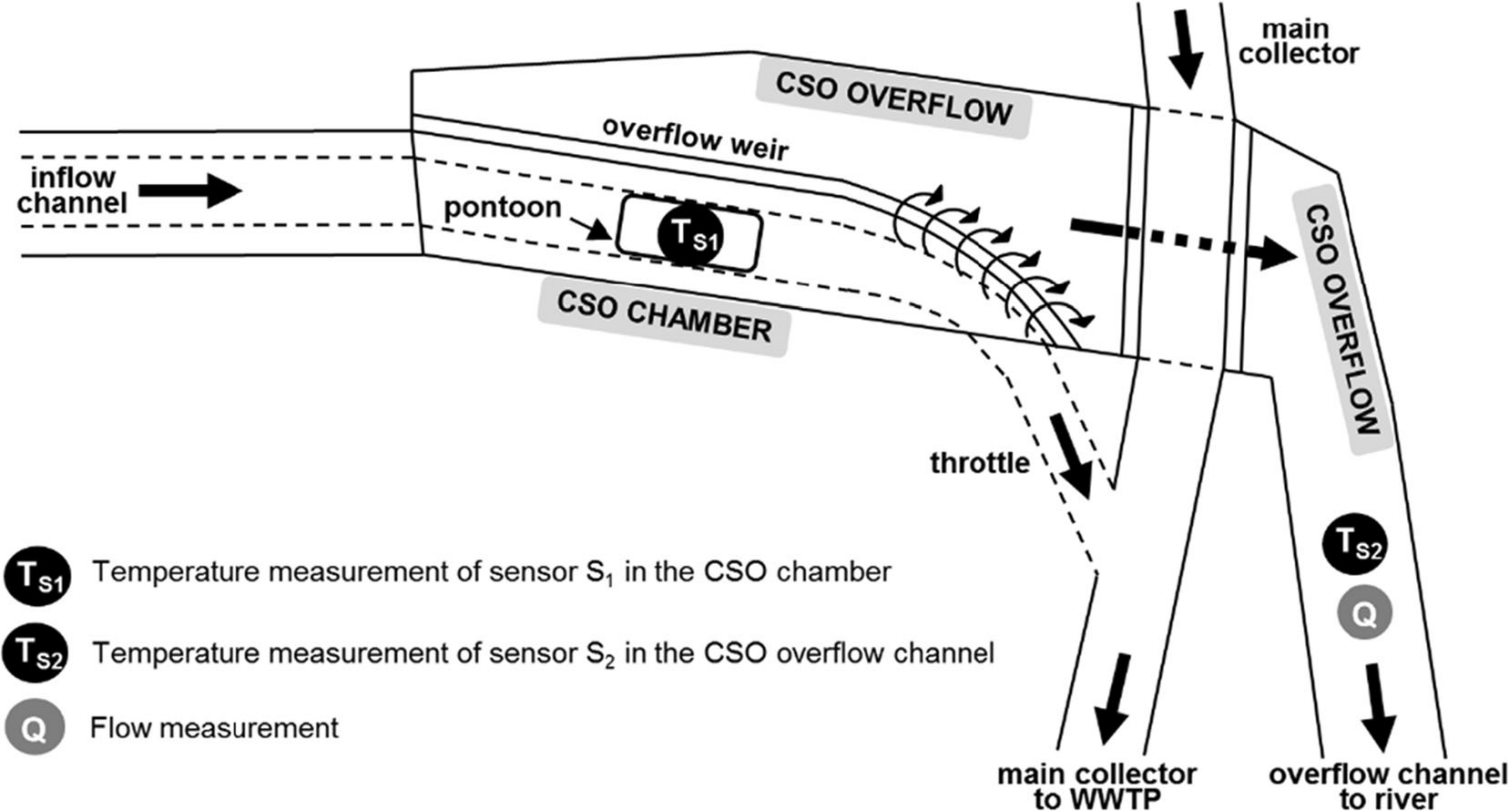
Experimental setup for CSO detection at a CSO structure in Graz (Austria)

To validate the developed methodology, an experimental setup in a CSO structure in Graz (Austria) was implemented. The CSO structure is equipped with several online monitoring devices and has been in operation since 2002 (Gruber et al. 2005; Gamerith et al. 2011). As shown in Fig. 2, the temperature sensor *S*_1_ (temperature signal *T*_S1_) was installed at the bottom of a floating pontoon in the CSO chamber to continuously measure the wastewater temperature. The temperature sensor *S*_2_ (temperature signal *T*_S2_) was installed on the invert of the CSO overflow channel to measure both the temperature of the ambient air during dry weather conditions and the temperature of overflowing wastewater during a CSO event. A previously installed flow measurement device in the CSO overflow channel (ultra-sonic flow meter) was used as a reference for CSO detection. For visual inspection and video recording, a waterproof webcam was directly installed in the CSO chamber.

Laboratory tests were performed to determine the sensor correction function *F*_SENSOR_(T) (refer to Eq. 1) separately for water and air conditions by reducing the temperature stepwise in intervals of 5 °C starting from 40 down to 5 °C. The measurement duration for each temperature level was defined with 10 min.

As a result, the relative deviation of the measured temperature signals was determined as a constant value of 0.1 °C for all of the analysed temperature levels for both water and air conditions, which leads to a constant sensor correction function of type *F*_SENSOR_(*T*) = 0.1.

The parameters of the time delay function *t*_DELAY_(*T*) were determined separately for water and air conditions (refer to Eq. 3). Two parameters, V (body volume) and A (heat transfer surface area), were calculated independently of the environmental conditions based on the known sensor geometry of *V* = 4.4 × 10^−5^ m^3^ and *A* = 1.9 × 10^−3^ m^2^. To determine the time delay function *t*_DELAY_(*T*) at the start of a CSO event (temperature change from air to overflowing wastewater), the density of wastewater was assumed to be equal to that of water, *ρ* = 1.0 × 10^6^ g^−3^. The isobaric mass heat capacity of water was assumed to be *c*_p_ = 4.1813 Jg^−1^ K^1^ based on literature (O’Connell and Haile 2005), and the heat transfer coefficient for water was determined from laboratory tests to be *h* = 800 Js^−1^m^−2^ K^−1^. The time constant τ was calculated to be *τ* = 121.0 s.

To determine the time delay function *t*_DELAY_(*T*) at the end of a CSO event (temperature change from overflowing wastewater to air), the density of air was approximated to *ρ* = 1.0 × 10^3^ g^−3^. The isobaric mass heat capacity of air was assumed to be *c*_p_ = 1.012 Jg^−1^ K^−1^ based on literature (O’Connell and Haile 2005), and the heat transfer coefficient for air was determined from laboratory tests to be *h* = 0.1 Js^−1^m^−2^ K^−1^. The time constant *τ* was calculated with *τ* = 234.4 s, which indicates a slower convergence behaviour for air compared to water conditions.

## Results and discussion

The CSO structure in Graz (Austria) was studied for a period of 7 months (from August 2012 to March 2013), in which a total of 20 CSO events occurred.

The start and end times as well as the durations of all CSO events detected by the reference method are presented in Table 3. The durations of the detected CSO events vary from 13 to 316 min. A graphical analysis of all detected CSO events is available as appendix in Online Resource 1.

**Table 3:** Detected CSO events by the reference method in the case study Graz (Austria) from August 05, 2012 to March 05, 2013

CSO event	Start time–end time	Duration	CSO event	Start time–end time	Duration
(#)	(date—dd.mm.yyyy hh:mm)	(min)	(#)	(date—dd.mm.yyyy hh:mm)	(min)
1	22.08.2012 20:17–22.08.2012 20:30	13	11	24.09.2012 20:18–24.09.2012 23:09	171
2	26.08.2012 08:30–26.08.2012 12:45	255	12	02.10.2012 03:03–02.10.2012 04:07	64
3	31.08.2012 11:43–31.08.2012 11:57	14	13	02.10.2012 08:10–02.10.2012 08:48	38
4	31.08.2012 17:11–31.08.2012 17:59	48	14	15.10.2012 18:49–15.10.2012 21:54	185
5	31.08.2012 21:06–01.09.2012 01:40	274	15	15.10.2012 23:16–16.10.2012 03:53	277
6	01.09.2012 18:56–01.09.2012 19:52	56	16	27.10.2012 16:31–27.10.2012 20:00	209
7	12.09.2012 19:35–12.09.2012 22:21	166	17	27.10.2012 21:35–27.10.2012 22:25	50
8	12.09.2012 23:41–13.09.2012 00:20	39	18	01.11.2012 04:55–01.11.2012 06:39	104
9	19.09.2012 11:02–19.09.2012 11:21	19	19	05.11.2012 04:37–05.11.2012 09:53	316
10	19.09.2012 17:31–19.09.2012 18:56	85	20	28.11.2012 21:00–28.11.2012 21:47	47

### Calibration results of the algorithm for the used type of temperature sensor

An initial calibration procedure was conducted to determine the optimum value of the CSO detection parameter Δ*T*_CSO_ for the used type of temperature sensor. Therefore, the results of a reference method (ultra-sonic flow meter, situated in the CSO overflow channel) and the proposed method were compared. For both methods the defined objective functions *P*_CSO,EVENTS_, *P*_CSO,TRUE_ and *P*_CSO,FALSE_ were calculated and compared. CSO detection by the reference method was defined by a threshold value of a 5 L/s flow rate, which leads to a detection of 20 CSO events in total during the observation period by using a defined CSO gap parameter *t*_GAP_ of 10 min.

Calibration of the CSO detection parameter Δ*T*_CSO_ was performed by calculating the values of the stated objective functions for a sequence of different values of Δ*T*_CSO_ from 0.1 up to 1.5 °C in an interval of 0.1 °C (minimum values for Δ*T*_CSO_ and intervals are defined by the temporal measurement resolution of the temperature sensor).

Based on the results of a previous sensitivity analysis, a number of five CSO events were determined as the minimum number of required CSO events for calibration. Using more CSO events for calibration always results in the same value for Δ*T*_CSO_, which indicates that no better calibration could be expected by using a higher number of CSO events.

A number of five consecutive CSO events (refer to CSO events 1–5 in Table 3) were used for calibration purposes. Table 4 shows a summary of all detected temperature matches *O* and all detected CSO events *N* for the investigated values of Δ*T*_CSO_. A total of 614 discrete CSO occurrences *O*_CSO,REF_, which is proportional to five detected CSO events *N*_CSO,REF_, was detected by the reference method. The detected temperature matches of the algorithm ranges from 606 (Δ*T*_CSO_ = 0.1 °C) to 1066 (Δ*T*_CSO_ = 1.5 °C). The number of CSO events detected by the algorithm ranges from 4 (Δ*T*_CSO_ = 0.1 °C) to 11 (Δ*T*_CSO_ = 1.5 °C).

**Table 4:** Calibration results of detected temperature matches *O* and CSO events *N* for different values of Δ*T*_CSO_

Δ*T*_CSO_	*O*_CSO,REF_	*O*_CSO_	*O*_CSO,TRUE_	*O*_CSO,FALSE_	*N*_CSO,REF_	*N*_CSO_
(°C)	(−)	(−)	(−)	(−)	(−)	(−)
0.1	614	606	601	5	5	4
0.2	614	613	608	5	5	5
0.3	614	611	606	5	5	5
0.4	614	619	606	13	5	5
0.5	614	627	608	19	5	6
0.6	614	636	611	25	5	6
0.7	614	642	609	33	5	6
0.8	614	650	610	40	5	6
0.9	614	668	609	59	5	6
1.0	614	701	611	90	5	8
1.1	614	743	609	134	5	8
1.2	614	823	610	213	5	8
1.3	614	881	609	272	5	9
1.4	614	949	610	339	5	10
1.5	614	1066	609	457	5	11

The values of the objective functions *P*_CSO,EVENTS_, *P*_CSO,TRUE_ and *P*_CSO,FALSE_ for the calibration process are presented in Table 5. The percentage of CSO events detected by the algorithm *P*_CSO,EVENTS_ = 100% shows that only for a range of Δ*T*_CSO_ = 0.2 to 0.4 °C all five CSO events were detected by the algorithm. With Δ*T*_CSO_ = 0.1 °C, only 80% of all events were detected, whereas with Δ*T*_CSO_ ≥ 0.5 °C, the number of events was always overestimated. The percentage of true positive temperature matches *O*_CSO,TRUE_ applying a progressively increasing Δ*T*_CSO_ showed values from 97.9 to 99.2%. In addition, the percentage of false positive temperature matches *O*_CSO,FALSE_ steadily increased from 0.8 to 74.74%.

**Table 5:** Calibration results of the objective functions *P*_CSO,EVENT_, *P*_CSO,TRUE_ and *P*_CSO,FALSE_ for different values of Δ*T*_CSO_

Δ*T*_CSO_	*P*_CSO,EVENTS_	*P*_CSO,TRUE_	*P*_CSO,FALSE_
(°C)	(%)	(%)	(%)
0.1	80.00	97.88	0.81
0.2	100.00	99.02	0.81
0.3	100.00	98.70	0.81
0.4	100.00	98.70	2.12
0.5	120.00	99.02	3.09
0.6	120.00	99.51	4.07
0.7	120.00	99.19	5.37
0.8	120.00	99.35	6.51
0.9	120.00	99.19	9.61
1.0	160.00	99.51	14.66
1.1	160.00	99.19	21.82
1.2	160.00	99.35	34.69
1.3	180.00	99.19	44.30
1.4	200.00	99.35	55.21
1.5	220.00	99.19	74.43

The best calibration results were achieved by using a value for Δ*T*_CSO_ of 0.2 °C, resulting in 608 true positive temperature matches of total 614 by the reference method and 5 false positive matches as well as the detection of all 5 CSO events. Related to the defined objective functions, the percentage of detected CSO events is 100%, and the ratios of true positive and false positive temperature matches are 99.02 and 0.81%, respectively.

### Validation results of the algorithm

Validation of the algorithm was performed by applying the determined value for the CSO detection parameter of Δ*T*_CSO_ = 0.2 °C to calculate the objective functions for a number of 15 independent CSO events (refer to CSO events 6–20 in Table 3).

Table 6 shows the validation results of all detected temperature matches *O* and all detected CSO events *N* for a value of Δ*T*_CSO_ = 0.2 °C. A total of 1882 discrete CSO occurrences *O*_CSO,REF_ were detected by the reference method, which is proportional to the 15 detected CSO events *N*_CSO,REF_. A number of 1873 temperature matches O_CSO_ were detected by the proposed method, resulting in 1834 of 1882 true positive temperature matches and 39 false positive temperature matches as well as the detection of all 15 CSO events.

**Table 6:** Validation results of detected temperature matches *O* and CSO events *N* for a value of Δ*T*_CSO_ = 0.2 °C

Δ*T*_CSO_	*O*_CSO,REF_	*O*_CSO_	*O*_CSO,TRUE_	*O*_CSO,FALSE_	*N*_CSO,REF_	*N*_CSO_
(°C)	(−)	(−)	(−)	(−)	(−)	(−)
0.2	1882	1873	1834	39	15	15

The values of the objective functions *P*_CSO,EVENTS_, *P*_CSO,TRUE_ and *P*_CSO,FALSE_ for the validation process by using Δ*T*_CSO_ = 0.2 °C are presented in Table 7. All of the CSO events used for validation could be detected (*P*_CSO,EVENTS_ = 100%). The percentage of true positive temperature matches *P*_CSO,TRUE_ is 97.5%, and the percentage of false positive temperature matches *P*_CSO,FALSE_ is 2.1%.

**Table 7:** Validation results of the objective functions *P*_CSO,EVENT_, *P*_CSO,TRUE_ and *P*_CSO,FALSE_ for a value of Δ*T*_CSO_ = 0.2 °C

Δ*T*_CSO_	*P*_CSO,EVENTS_	*P*_CSO,TRUE_	*P*_CSO,FALSE_
(°C)	(%)	(%)	(%)
0.2	100.00	97.45	2.07

Related to the defined objective functions, the percentage of detected CSO events is 100%, and the ratios of true positive and false positive temperature matches are 97.5 and 2.1%, respectively.

### Results considering time delay correction

**Fig. 3:**
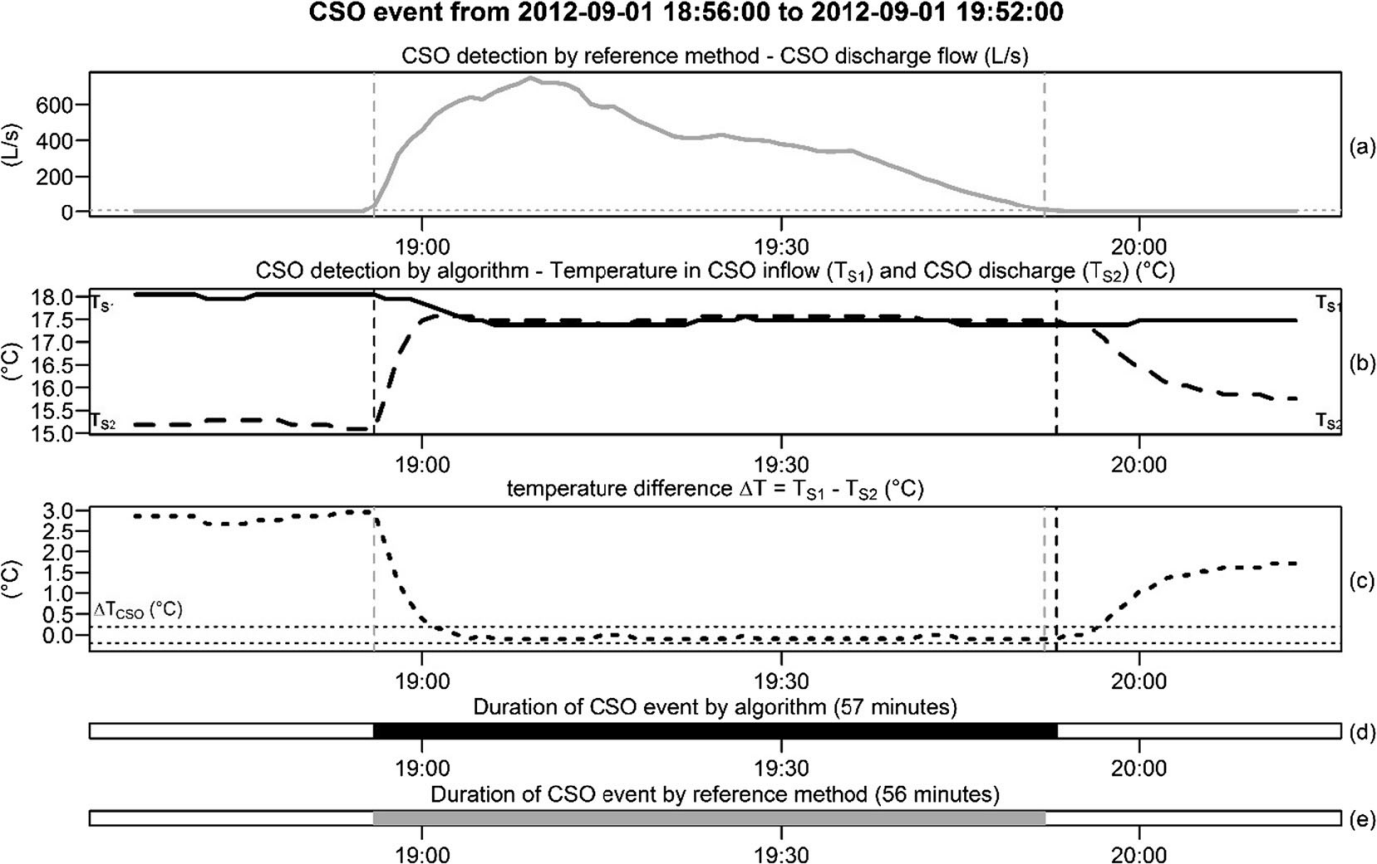
Graphical analysis of the detected CSO event #6: comparison of the developed algorithm and reference method

Figure 3 shows a graphical analysis of CSO event #6, which was detected by the reference method on September 01, 2012, from 18:56 to 19:52, with a duration of 56 min.

Figure 3a shows the measured flow rates in the CSO overflow channel. The start and end of the CSO event detected by the reference method are marked by grey vertical dashed lines. The pre-defined threshold value for a CSO detection of 5 L/s is marked as a grey horizontal dashed line.

Figure 3b shows the temperature signals *T*_S1_ in the CSO chamber (black solid line) and *T*_S2_ in the CSO overflow channel (black dashed line).

Figure 3c shows the difference between the temperature signals *T*_S1_ and *T*_S2_. The black horizontal dashed lines indicate the CSO detection parameter Δ*T*_CSO_ = ± 0.2 °C, and the black vertical dashed lines mark the start and end of the CSO event detected by the algorithm. The start and end of the detected CSO event detected by the reference method are marked as grey vertical dashed lines for comparison.

The CSO event detected by the algorithm starts at exactly the same time and ended 1 min later for a duration of 57 min (the reference method had a duration of 56 min; see Fig. 3d, e).

**Fig. 4:**
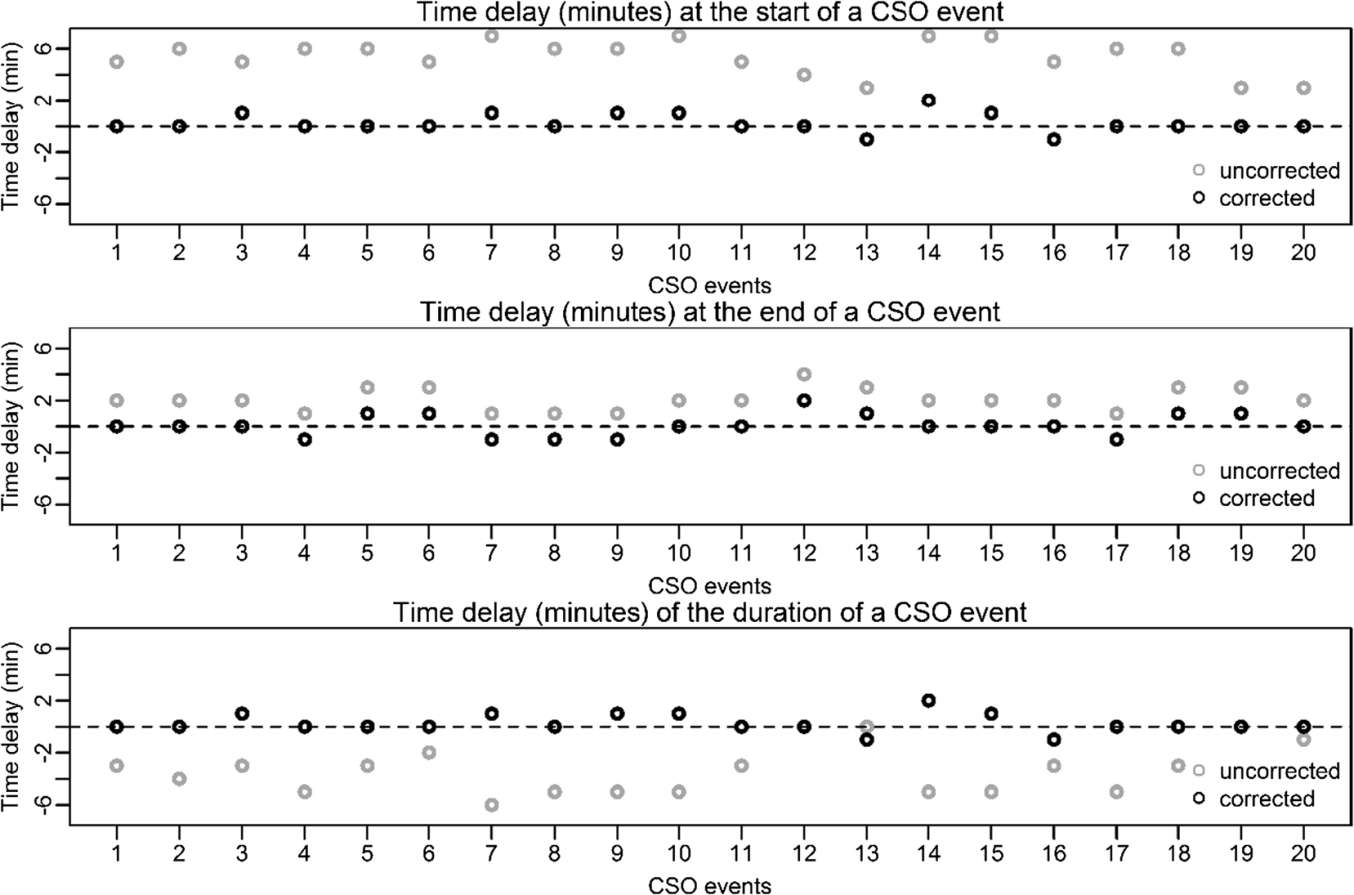
Analysis of time delays for the start, the end and the duration of detected CSO events (uncorrected time delay = grey; corrected time delay = black)

A detailed analysis of the observed time delay in the detection of the algorithm and its correction is presented in Fig. 4. Detection values of time delay without correction are marked as grey points, and values with time delay correction are marked as black points. Without correction, all time delays at the start and the end of a CSO event have positive values, which indicate that, in this case, start and end of all detected CSO events are always later compared to the reference method. Without correction, maximum deviations of 7 min (start) and 4 min (end) are obtained. The resulting deviations in the duration of the detected CSO events are always negative, which indicates shorter CSO event durations by the reference method. Without correction, maximum deviations in the duration of CSO events of − 6 min are obtained. With time delay correction, maximum deviations of 2 min (start), 2 min (end) and 2 min (duration) are obtained.

### Application of the method

The presented method for CSO detection is capable of estimating occurrence and duration of CSO events with very simple hardware (low-cost temperature sensors) and a more advanced software (the corresponding algorithm).

The method proved robust, detecting 20 CSO events without any false detection during the investigation period. An optimum value of 0.2 °C for the CSO detection parameter Δ*T*_CSO_ was identified for the applied type of temperature sensor through the calibration and validation process, which leads to a maximum percentage of true positive temperature matches (99.02%) and a minimum percentage of false positive temperature matches (0.81%) (Table 5). The maximum deviation in CSO duration compared to the reference method was 2 min.

The calibrated algorithm is generally transferable to other CSO structures provided that the same type of temperature sensor is used. An application of a different type of temperature sensor with divergent specifications in accuracy, resolution, stability and time-synchronicity requires a new calibration process, which is structured in four steps. The first step is the determination of the sensor correction function *F*_SENSOR_(*T*) in the lab with the accuracy of the sensor type (°C) as only influence factor. The overall accuracy is of lower importance than the relative consistency of the sensor signals. The second step of the calibration process is the determination of the time delay correction function *t*_DELAY_(*T*) in the lab. Its influence factor is the thermal response time of the sensor characterized by the density *ρ* of the medium (gm^−3^), the isobaric mass heat capacity cp (Jg^−1^ K^−1^), the body volume *V* (m^3^), the heat transfer coefficient *h* (Js^−1^m^−2^K^−1^) and the heat transfer surface area *A* (m^2^). A fast response time of the sensor of 5 min or less is recommended. The third step is the definition of the CSO gap parameter *t*_GAP_, which is influenced only by the flow dynamics in the CSO structure and is independent of the sensor type. In this study, a value of 10 min was suitable for CSO detection. The fourth step determines the CSO detection parameter Δ*T*_CSO_ against a reference CSO detection method inside the CSO structure. This parameter is characterized by the sensor specifications like resolution, stability and time-synchronicity. In this study, the optimal value of this parameter was determined by 0.2 °C. The related sensor specifications were 0.14 °C for resolution, 0.1 °C per year for stability and ± 1 min per month for time-synchronicity.

The transfer of the presented method for a calibrated sensor type to other monitoring locations is possible without further calibration efforts.

The applied ultra-sonic flow measurements as a reference method have been successfully used to validate the presented method. If enough data are available, the reference dataset can be split into a training period in which a sufficient number of CSO events occur. The quality of the calibration can be quantified during the validation period by calculating defined objective functions, which are based on the detection rate of the algorithm. Sensitivity analyses showed that five CSO events were sufficient to determine the CSO detection parameter Δ*T*_CSO_. Using a lower number of events introduces some uncertainty in defining the CSO detection parameter, whereas a higher number has no further influence on the final parameter value.

### Advantages and limitations of the method

The CSO detection method has been designed to minimize false CSO detections. These false detections may occur under dry weather conditions when air and wastewater temperatures inside the CSO structure reach equal values. This situation might happen due to (i) natural temperature variability (e.g. increased air temperature in summer time), (ii) due unexpected discharges in the sewer system, (iii) incomplete submersion of temperature sensor *T*_1_ inside the wastewater stream, (iv) a partial or complete submersion of temperature sensor *T*_2_ by backwater effects from the receiving water body.

This study shows that the proposed method is robust against false detections for the following two reasons. The first reason is the defined minimum CSO detection duration of 10 min. Therefore, potential short-term convergences do not lead to false detections. The second reason is the CSO detection parameter Δ*T*_CSO_ of 0.2 °C, which is a precise value to determine the convergence of the temperature signals. False detections seem to be improbable regarding to the variable temperature pattern of domestic wastewater inside a combined sewer system. The monitoring period in this study of 7 months from August to March included seasonal periods of summer, autumn and winter without false detections.

It is recommended to place temperature sensor *T*_1_ inside the wastewater stream in a way that it is at any time completely submerged. Sensor *T*_2_ can be placed directly on top of the overflow weir crest or at the invert of the overflow channel. Sensor *T*_2_ should not be installed in the airspace behind the overflow wave, in depressions on the invert or in the area of the overflow channel which can be flooded with water from the river due to backwater effects.

The minimum duration of 10 min for detectable CSO events is depending on the temperature difference of the two temperature measurements *T*_S1_ and *T*_S2_. This is caused by the physical inertia of the heat transfer from the water or gas environment to the sensor and vice versa, which can be described by Newton’s law of cooling. If the duration of the overflow event is shorter than the time needed by the sensor to adapt to the changed temperature, it is not possible to reliably detect the occurring CSO event. Therefore, a temperature match for at least one-time step should be obtained. This is not a significant limitation compared to other surrogate methods, even compared to the reference method used in this study. Most of these methods are uncertain for detecting small overflow events. Additionally, some methods have problems regarding to false detections, which could be avoided completely with the presented method.

A potential risk exists that the sensors in the CSO chamber are clogged with sanitary products or the sensors are covered with sewer sediments. Due to the small size of the used sensors, different methods of installation can easily be applied to reduce these risks.

The used type of sensor was robust in operation for the entire duration of 7 months in the study. The sensors did not show any measurement failures caused by changing conditions during summer and winter period or caused by CSO events. The chosen maintenance frequency of 1 month was sufficient to prevent sensor clogging or fouling.

An advantage of the used temperature sensors is their independent battery power supply with a durability of 1 year. Therefore, no extra cabled power supply is required on site, which reduces investment and operational costs.

Legislation in some countries stipulates that sensors and other electronic equipment with voltage supply higher than 12 V installed directly in sewer systems have to be designed to be explosion-proof. The temperature sensors employed in this study use a low battery voltage supply of 3 V, which enables their application in sewer systems without explosion-proof requirements.

Data retrieval in this study was performed manually. This implies a demand on man power, which is costly and time consuming and also implies safety issues. If it is not possible to enter the CSO structure periodically, or if several CSO structures should be monitored, additional compatible equipment for automatic data transmission (e.g. Bluetooth, Wi-Fi, GSM, etc.) is desirable.

### Comparison with already existing methods

For an objective evaluation of the characteristics of the introduced methods, a comparison with already existing methods for CSO detections is essential. The comparison is summarized in Table 8 by the following criteria:

- CSO occurrence, duration and volume
- CSO detection rate
- Costs
- Required information
- Effort
- Transferability

**Table 8:** Comparison with already existing methods for CSO detection

CSO occurrence, duration and volume		
Presented method	Existing methods	
The method is able to detect occurrence and duration of a CSO event with an accuracy of 2 min.	Similar methods: (a), (g), (h), (j), (l), (m), (n), (o) Worse methods: (c)–(f) Better methods: (b), (i), (k)	
CSO detection rate		
Presented method	Existing methods	
The method has a CSO detection rate of 100% out of 20 CSO events.	Method (h) stated a CSO detection rate of 90% out of 168 CSO events. Method (o) stated a CSO detection rate of 80% out of 57 rainfall events.	
Costs		
Presented method	Existing methods	
The method has invest costs excluding data transmission of about 100 € per CSO structure.	Similar methods: (e), (f), (l), (n), (o) Cheaper methods: none Dearer methods: (a)–(d), (g)–(k), (m)	
Required information		
Presented method	Existing methods	
The method requires no advance information for application using the described sensor type.	Similar methods: (a), (b), (l), (n), (o) Low information need: (c)–(f) High information need: (g)–(k), (m)	
Effort		
Presented method	Existing methods	
The method has low effort in installation, operation and data evaluation. Manual data retrieval represents a higher effort. The overall effort is low.	Similar methods: (a), (c), (d), (o) Lower effort: (n) Higher effort: (b), (e)–(m)	
Transferability		
Presented method	Existing methods	
The transferability of the method is high, including all criteria from above.	Similar methods: (a), (n), (o) Average transferability: (b)–(d), (i), (j) Low transferability: (e)–(h), (k)–(m)	
(a) Rasmussen et al. (2008)	(b) Sonnenberg et al. (2011)	(c) Schilperoort et al. (2006)
(d) Riechel et al. (2010)	(e) Weyrauch et al. (2010)	(f) Buerge et al. (2006)
(g) Thorndahl and Willems (2008)	(h) Schroeder et al. (2011)	(i) Jeanbourquin et al. (2011)
(j) Lo et al. (2015)	(k) Yu et al. (2013)	(l) Siemers et al. (2011)
(m) Gruber et al. (2005)	(n) Wani et al. (2017)	(o) Montserrat et al. (2013)

Most of the available surrogate methods have the same CSO detection ability (CSO occurrence and CSO duration) as the proposed method. All detection methods based on measurements in the receiving water body are only able to detect CSO occurrence. The CSO volume is only detectable by common flow measurements (Sonnenberg et al. 2011), video image analysis (Jeanbourquin et al. 2011) and model cluster analysis (Yu et al. 2013).

In the majority of cases the described methods in literature provide no values for CSO detection rate. Only Schroeder et al. (2011) and Montserrat et al. (2013) provide detection rates. Both methods have a lower detection rate than the proposed method.

All described methods are more or equally expensive compared to the proposed method.

Provided that the described or comparable sensors in respect of accuracy, resolution, stability and time-synchronicity are applied, the proposed method and five other methods need no advance information for application at CSO structures. The majority of methods require pre-information about specific measurement data or simulation models. The overall effort of the proposed method is low regarding installation, operation, data retrieval and data evaluation. The manual data retrieval process can be carried out during a monthly inspection and the script-based data evaluation requires basic skills in a mathematical computing software environment or programming languages like R (R Core Team 2017), Scilab (Scilab enterprises 2017) or Matlab (MathWorks 2017).

Except of CSO detection based on flow or water level (Sonnenberg et al. 2011), all other methods require a script-based data evaluation based on different programming languages.

The transferability of the stated methods is comparable with the methods using electrical contacts (Rasmussen et al. 2008), moisture sensors (Wani et al. 2017) and a single low-cost temperature sensor (Montserrat et al. 2013). All other methods have a lower potential of transferability regarding economic aspects.

## Conclusions

The application of two temperature sensors installed inside a CSO structure was proven to be a robust and accurate surrogate method for the automatic detection of the occurrence and duration of CSO events. Within the 7-month test phase, all 20 CSO events could be detected without false detections.

For the used type of temperature sensor, an optimum value of 0.2 °C for the CSO detection parameter Δ*T*_CSO_ achieved the best results in detection. A parameter variation in the range of 0.1 to 1.5 °C has shown that a value of 0.2 °C leads to a maximum percentage of true positive temperature matches and a minimum percentage of false positive temperature matches, which enables the detection all CSO events compared to conventional ultra-sonic flow measurements without false detections.

The accuracy of detecting the start and end of the CSO events was 2 min in comparison to the reference method.

Compared to existing approaches for CSO detection the proposed approach has a higher detection rate compared to other methods. The invest costs are low and no preconditions or advance information are required using the stated sensors. The overall effort is low and the transferability regarding economic aspects to other CSO structures is high.

The proposed method can be used at different monitoring locations using the same settings as long as the same types of temperature sensors are used. The transfer of the proposed methodology to other CSO structures is easily possible. This transferability offers a cost-effective opportunity to simultaneously monitor several CSOs in a sewer system at the same time. The spatially distributed information on duration of CSO events can be used for the calibration and validation of sewer simulation models (Montserrat et al. 2017; Wani et al. 2017).

A possibility to further increase the detection rate of the method could be the integration of a rain gauge signal as an additional trigger for a CSO event. A simple low-cost system would be sufficient to detect if it is raining or not, since an exact measurement of the precipitation intensity is not absolutely necessary.

The method could be extended to have the capacity to detect the presence of water in the sewer system coming from the river caused by backwater effects. Such extension is based on the addition of an extra temperature sensor directly submerged in the river at the CSO outlet.

A potential risk of clogging the sensors with hygienic articles or covering the sensors with sewer sediment exists. Due to the small size and weight of the sensors, installation techniques to reduce this risk can easily be applied. In operation, a monthly inspection of the sensors is recommended and can be combined with the data retrieval process.

Using low-cost temperature sensors instead of common measurement systems for CSO monitoring has the potential to significantly decrease the purchasing and operational costs of local sewer operators.

## Electronic supplementary material

ESM 1 (DOCX 3804 kb)
