# Supplementary material for: A robust and accurate surrogate method for monitoring the frequency and duration of combined sewer overflows
Source: Environ Monit Assess. 2018 Mar 11;190(4):209. doi: 10.1007/s10661-018-6589-3 (PMC5846818; doi:10.1007/s10661-018-6589-3)
Supplement: Supplementary file 1 — (DOCX 3804 kb) [file 10661_2018_6589_MOESM1_ESM.docx]

A robust and accurate surrogate method for monitoring the frequency and duration of combined sewer overflows

Thomas Hofer^1*^, Albert Montserrat^2^, Guenter Gruber^1^, Valentin Gamerith^1,3^, Lluis Corominas^2^, Dirk Muschalla^1^

^1^ Graz University of Technology, Institute of Urban Water Management and Landscape Water Engineering,
Stremayrgasse 10/I, 8010 Graz, Austria.

^2^ Catalan Institute for Water Research, Scientiﬁc and Technological Park of the University of Girona,
H2O Building, Emili Grahit 101, 17003 Girona, Spain.

^3^ Hydroconsult GmbH, Engineering Company for Environmental Engineering and Water Management,
St. Veiter Straße 11a, 8045 Graz, Austria.

*Corresponding author: thomas.hofer@tugraz.at, Tel.: +43 316 873 6768; Fax: +43 316 873 8376

**Appendix**

Table A1 Detected CSO events by the reference method in the case study Graz (Austria) from August 05, 2012 to
March 05, 2013

| **CSO event** | **start time – end time** | **duration** | **CSO event** | **start time – end time** | **duration** |
| --- | --- | --- | --- | --- | --- |
| **(#)** | **(date - dd.mm.yyyy hh:mm)** | **(min)** | **(#)** | **(date - dd.mm.yyyy hh:mm)** | **(min)** |
| **1** | 22.08.2012 20:17 - 22.08.2012 20:30 | 13 | **11** | 24.09.2012 20:18 - 24.09.2012 23:09 | 171 |
| **2** | 26.08.2012 08:30 - 26.08.2012 12:45 | 255 | **12** | 02.10.2012 03:03 - 02.10.2012 04:07 | 64 |
| **3** | 31.08.2012 11:43 - 31.08.2012 11:57 | 14 | **13** | 02.10.2012 08:10 - 02.10.2012 08:48 | 38 |
| **4** | 31.08.2012 17:11 - 31.08.2012 17:59 | 48 | **14** | 15.10.2012 18:49 - 15.10.2012 21:54 | 185 |
| **5** | 31.08.2012 21:06 - 01.09.2012 01:40 | 274 | **15** | 15.10.2012 23:16 - 16.10.2012 03:53 | 277 |
| **6** | 01.09.2012 18:56 - 01.09.2012 19:52 | 56 | **16** | 27.10.2012 16:31 - 27.10.2012 20:00 | 209 |
| **7** | 12.09.2012 19:35 - 12.09.2012 22:21 | 166 | **17** | 27.10.2012 21:35 - 27.10.2012 22:25 | 50 |
| **8** | 12.09.2012 23:41 - 13.09.2012 00:20 | 39 | **18** | 01.11.2012 04:55 - 01.11.2012 06:39 | 104 |
| **9** | 19.09.2012 11:02 - 19.09.2012 11:21 | 19 | **19** | 05.11.2012 04:37 - 05.11.2012 09:53 | 316 |
| **10** | 19.09.2012 17:31 - 19.09.2012 18:56 | 85 | **20** | 28.11.2012 21:00 - 28.11.2012 21:47 | 47 |

**
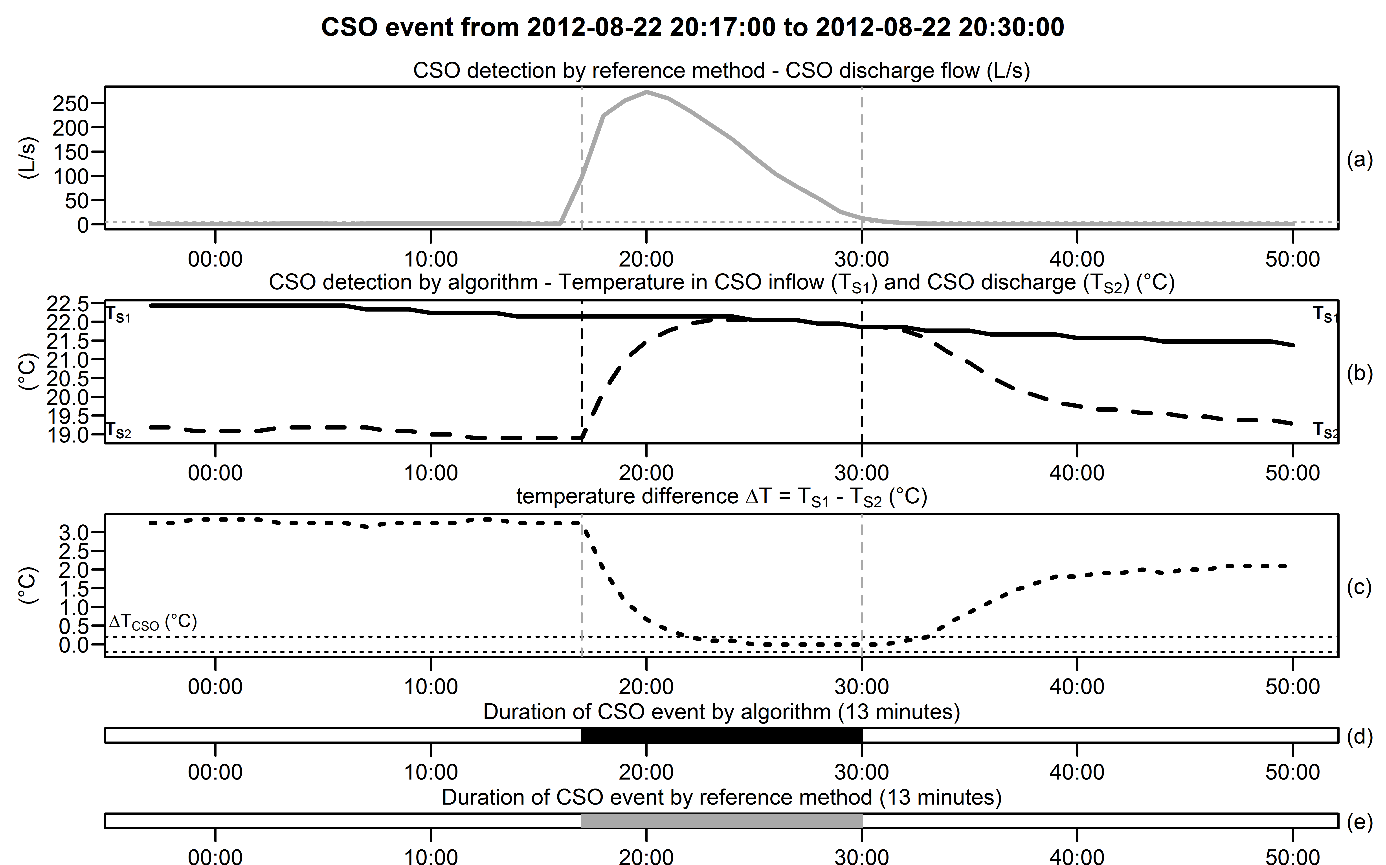
**

**Fig. A1** Graphical analysis of the detected CSO event #1 (comparison of developed algorithm and reference method) from 2012-08-22 20:17 to 2012-08-22 20:30


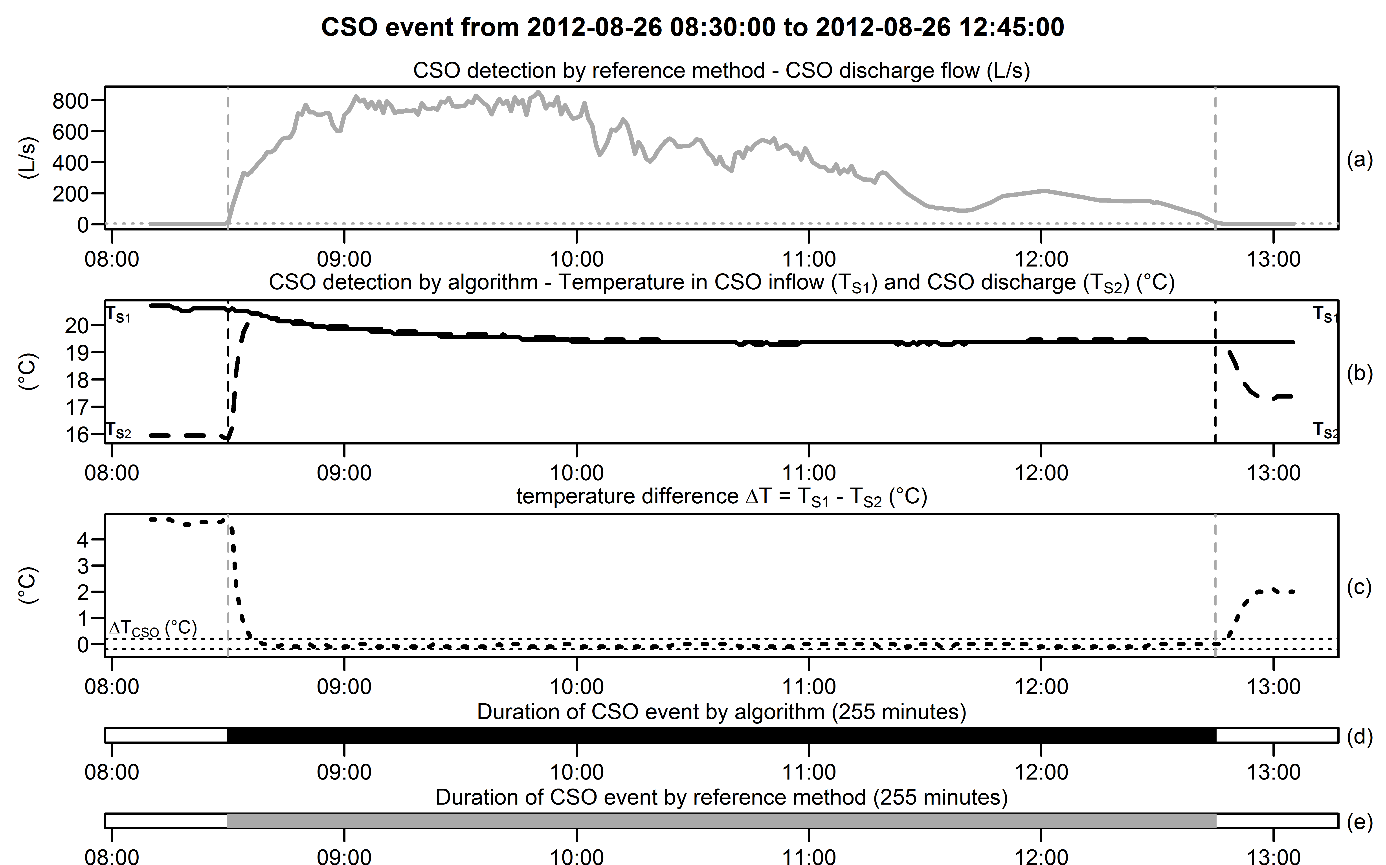


**Fig. A2** Graphical analysis of the detected CSO event #2 (comparison of developed algorithm and reference method) from 2012-08-26 08:30 to 2012-08-26 12:45.


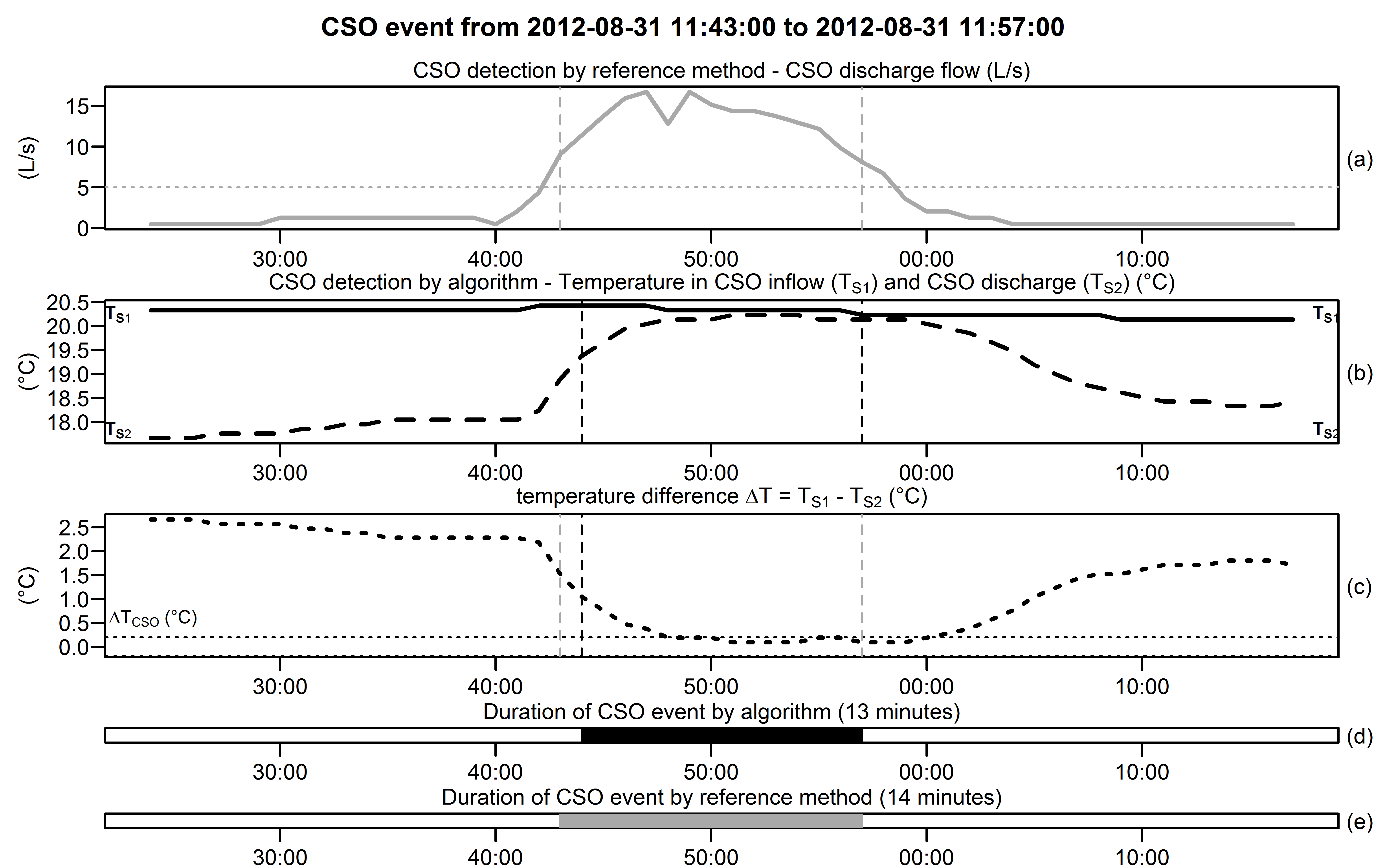


**Fig. A3** Graphical analysis of the detected CSO event #3 (comparison of developed algorithm and reference method) from 2012-08-31 11:43 to 2012-08-31 11:57.


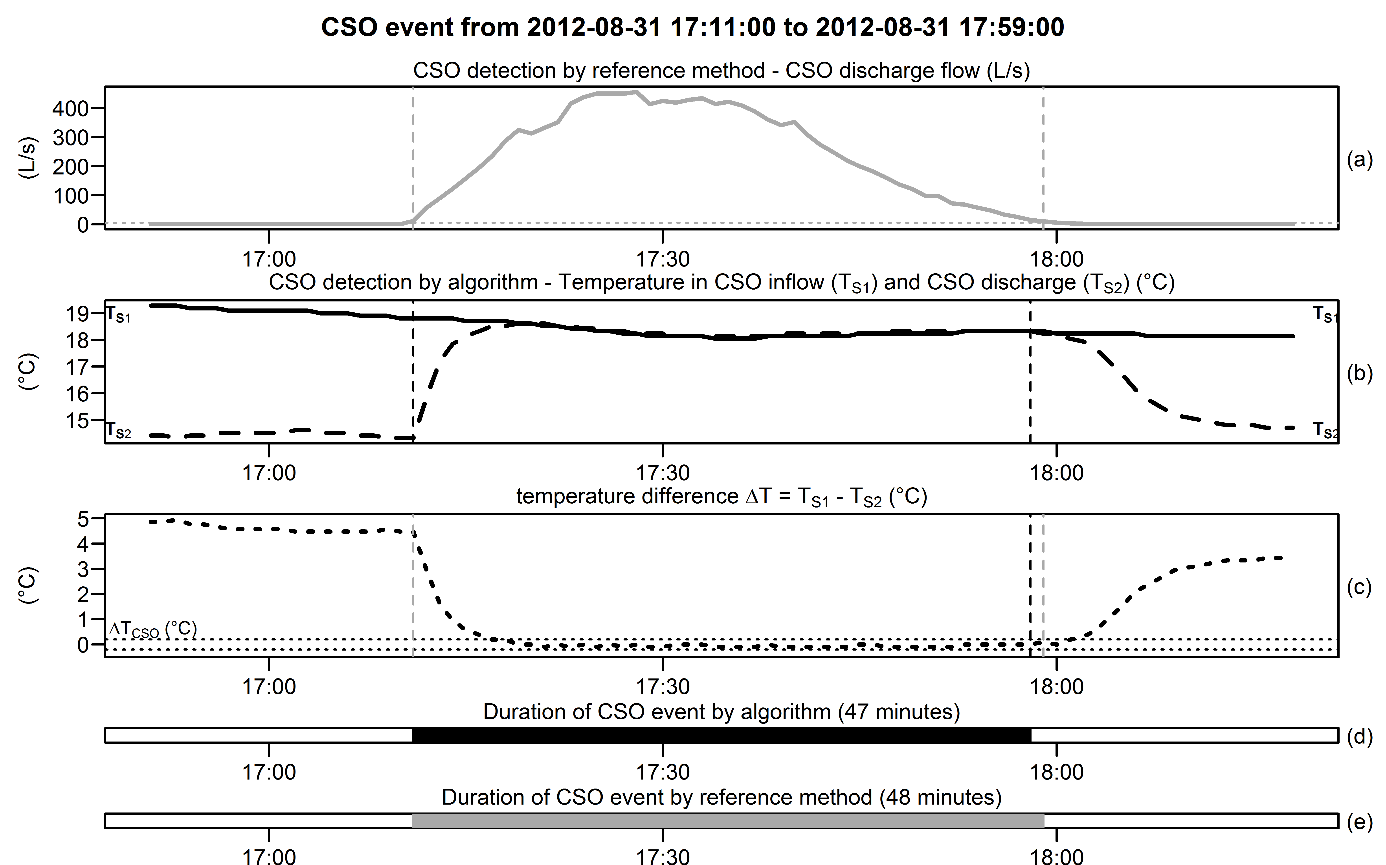


**Fig. A4** Graphical analysis of the detected CSO event #4 (comparison of developed algorithm and reference method) from 2012-08-31 17:11 to 2012-08-31 17:59.


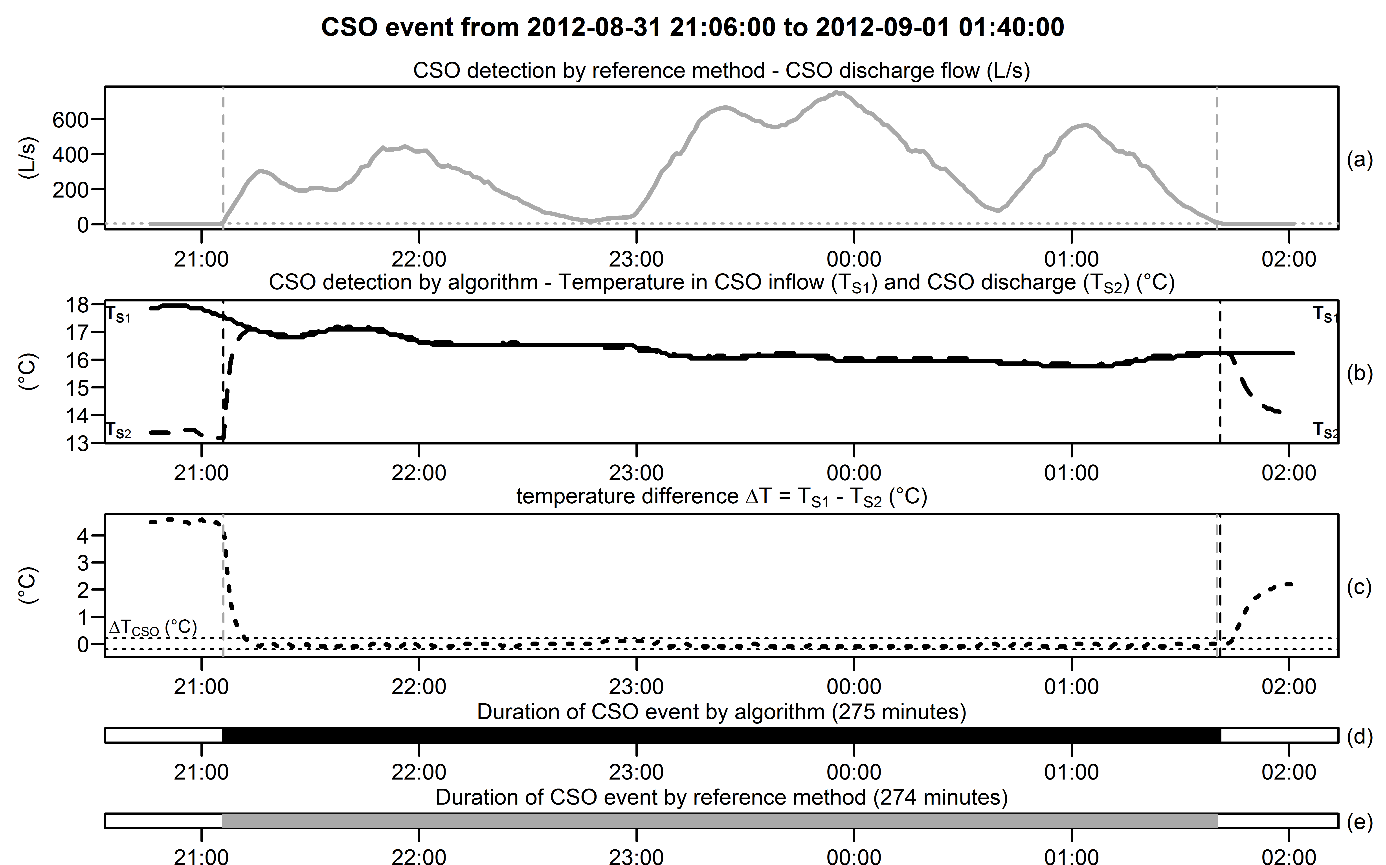


**Fig. A5** Graphical analysis of the detected CSO event #5 (comparison of developed algorithm and reference method) from 2012-08-31 21:06 to 2012-09-01 01:40.


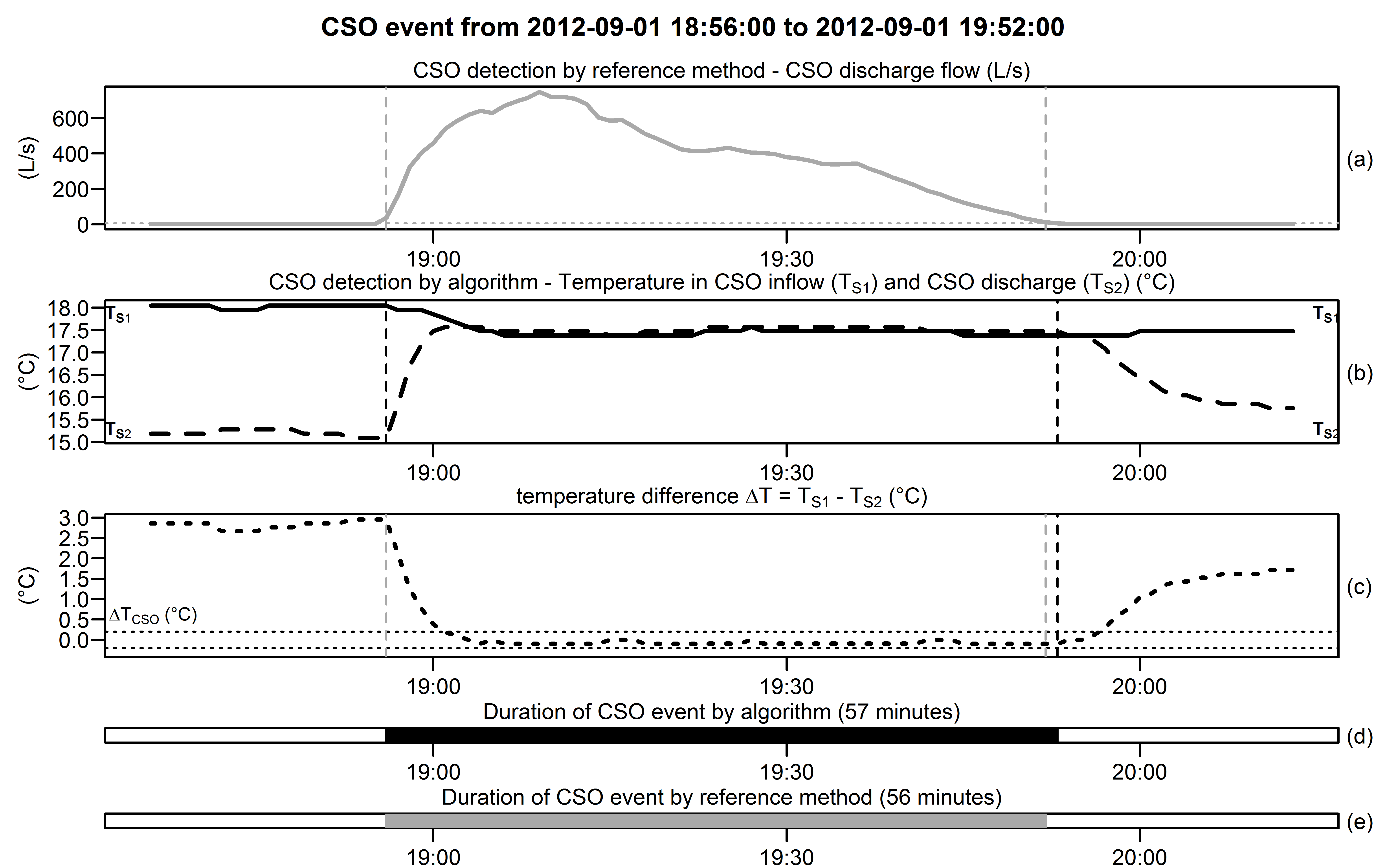


**Fig. A6** Graphical analysis of the detected CSO event #6 (comparison of developed algorithm and reference method) from 2012-09-01 18:56 to 2012-09-01 19:52.


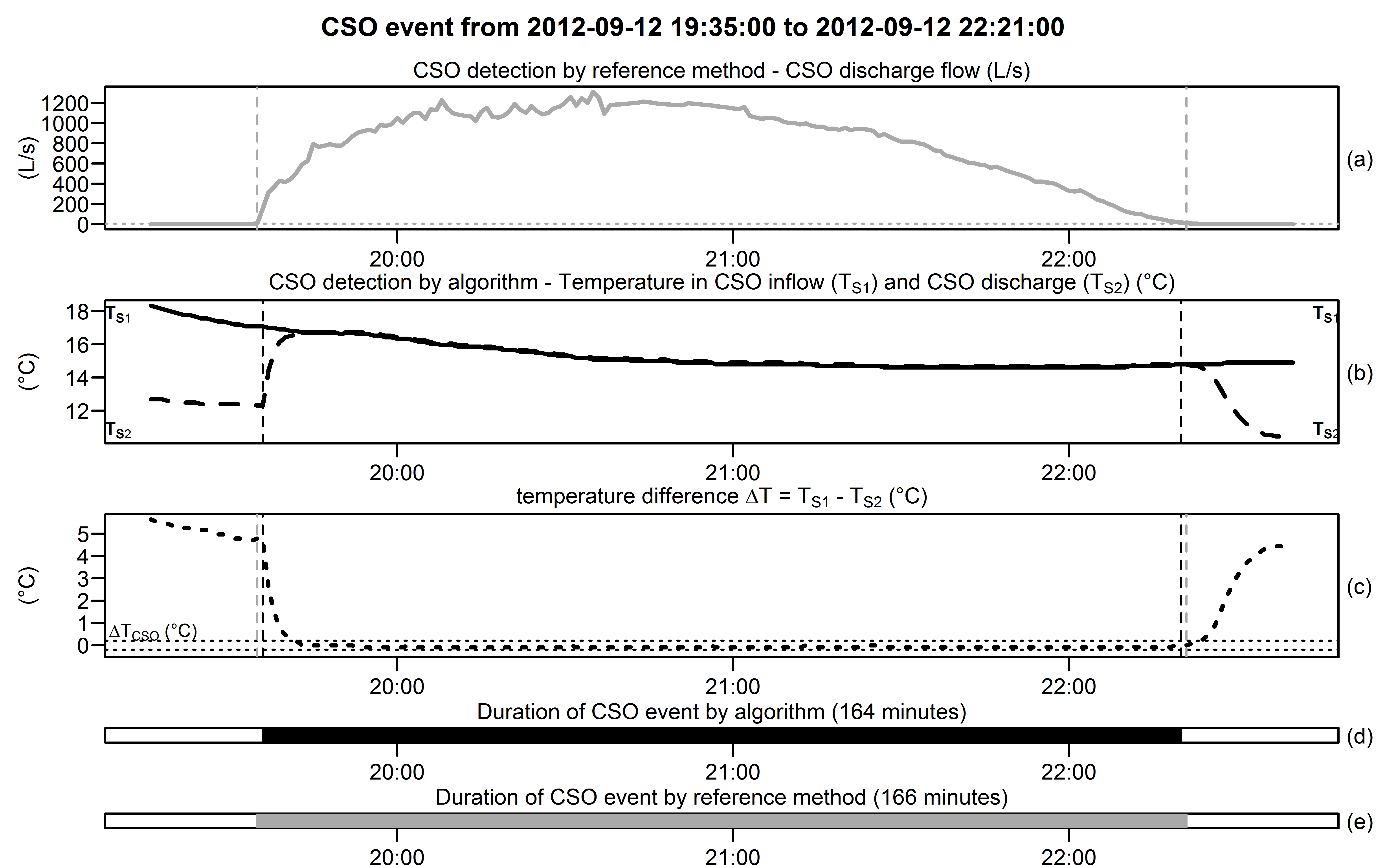


**Fig. A7** Graphical analysis of the detected CSO event #7 (comparison of developed algorithm and reference method) from 2012-09-12 19:35 to 2012-09-12 22:21.


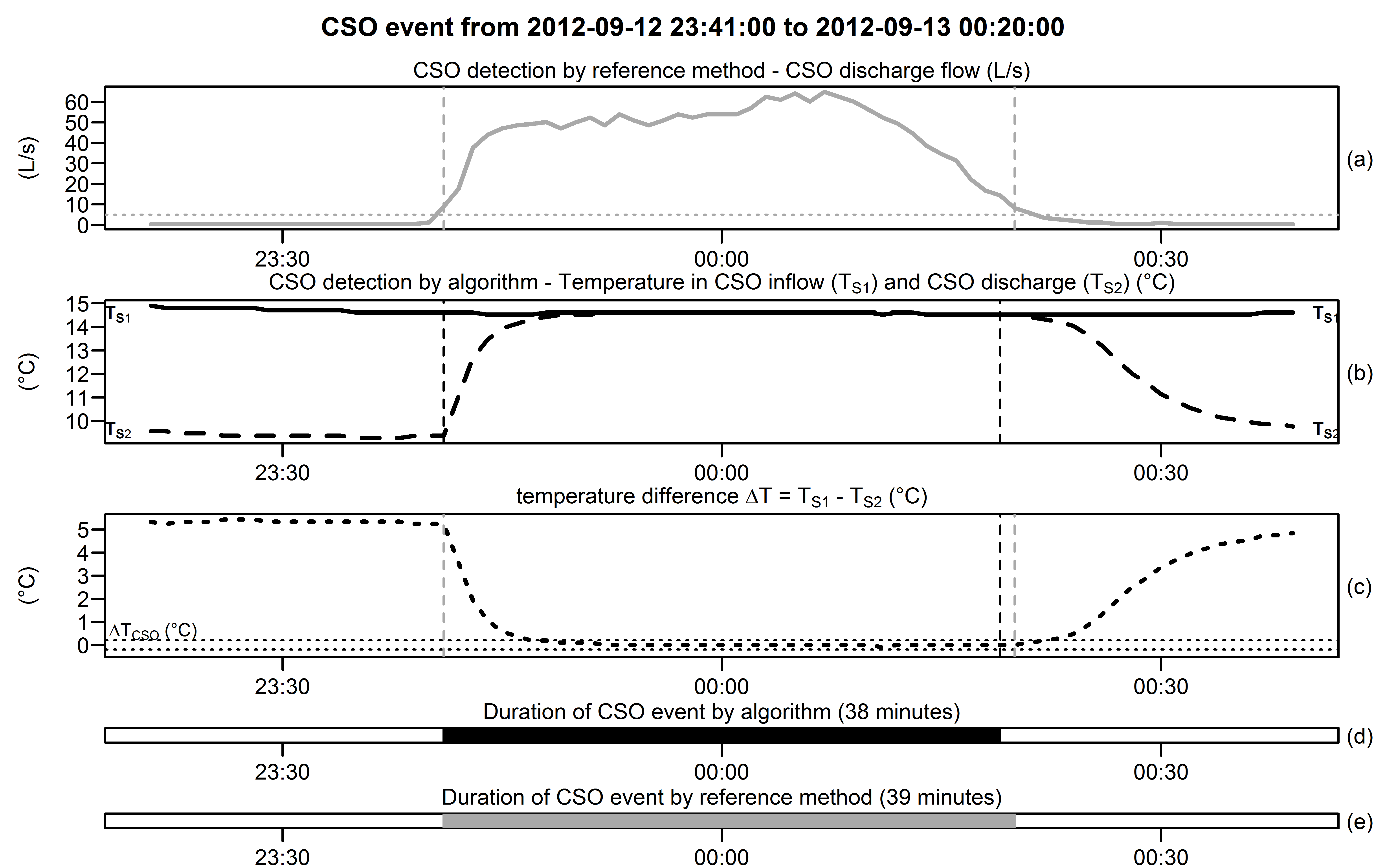


**Fig. A8** Graphical analysis of the detected CSO event #8 (comparison of developed algorithm and reference method) from 2012-09-12 23:41 to 2012-09-13 00:20.


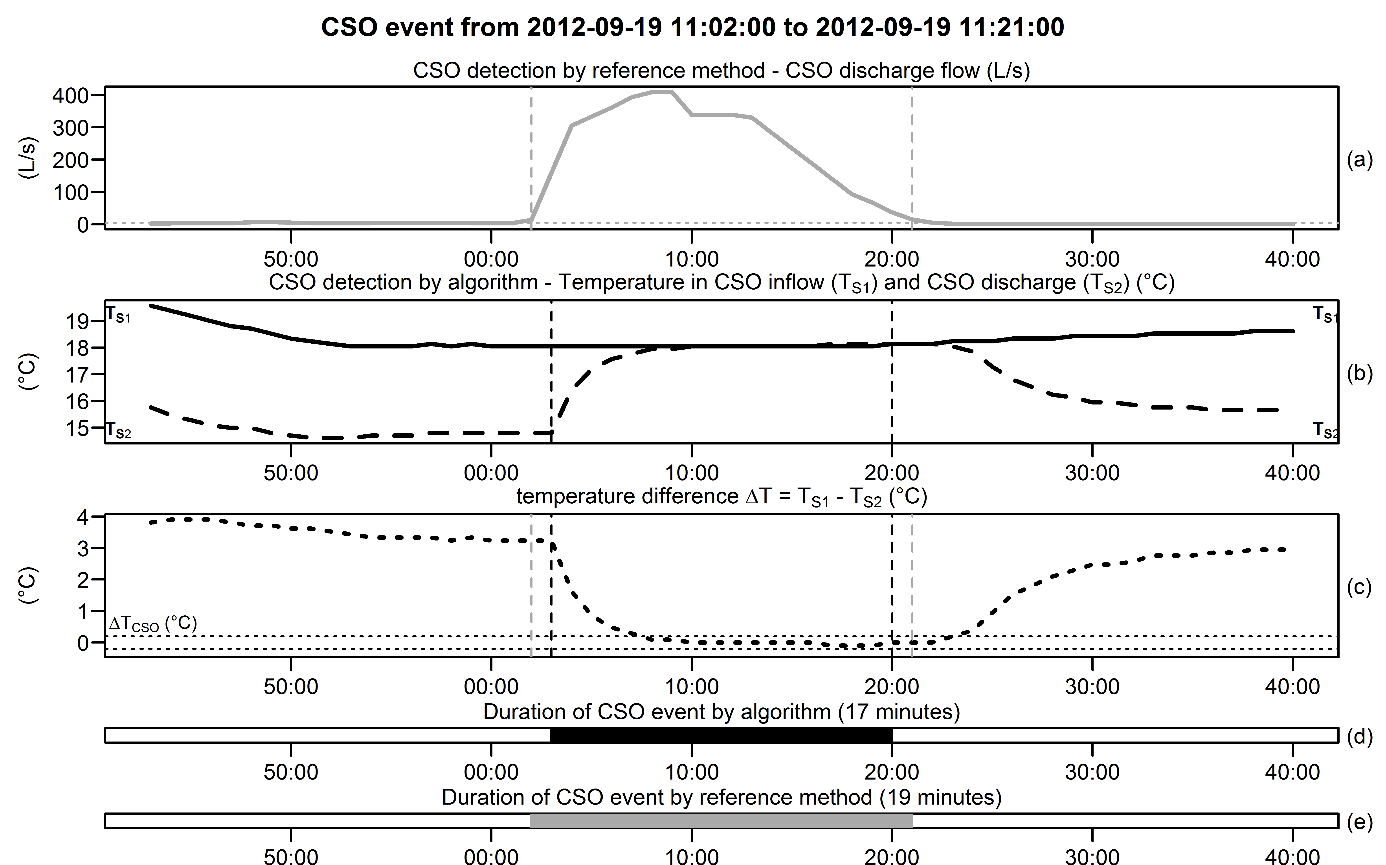


**Fig. A9** Graphical analysis of the detected CSO event #9 (comparison of developed algorithm and reference method) from 2012-09-19 11:02 to 2012-09-19 11:21.


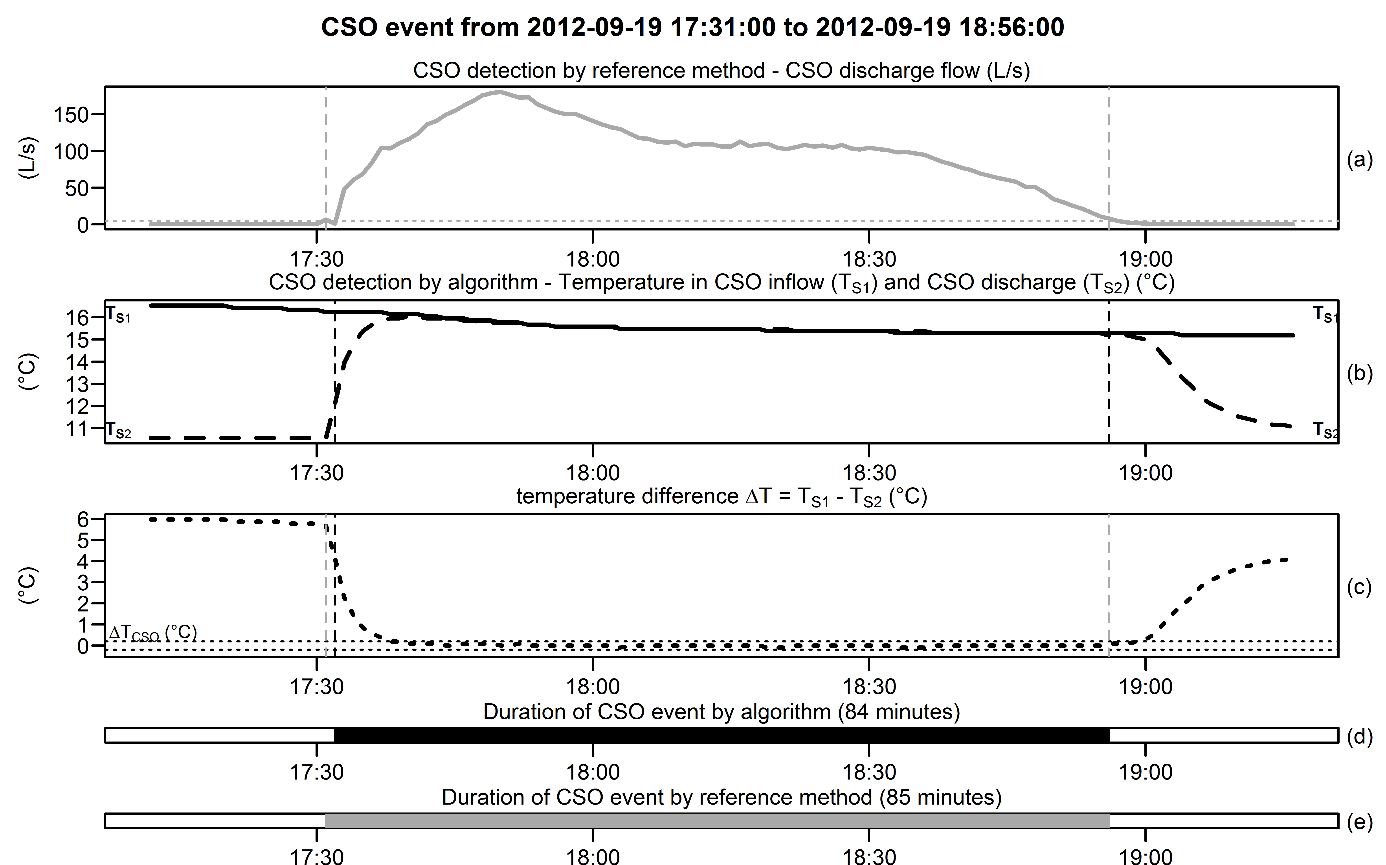


**Fig. A10** Graphical analysis of the detected CSO event #10 (comparison of developed algorithm and reference method) from 2012-09-19 17:31 to 2012-09-19 18:56.


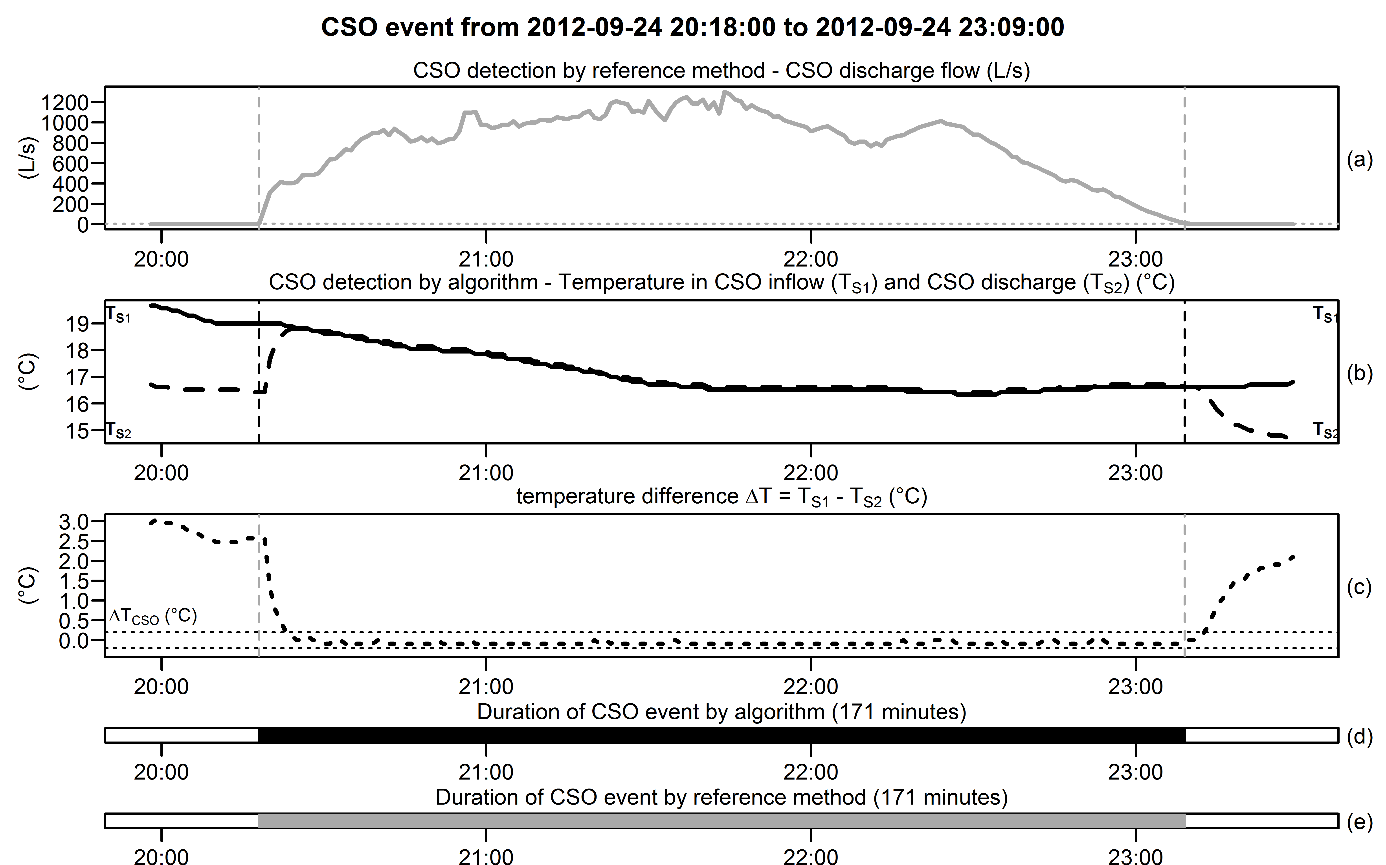


**Fig. A11** Graphical analysis of the detected CSO event #11 (comparison of developed algorithm and reference method) from 2012-09-24 20:18 to 2012-09-24 23:09.


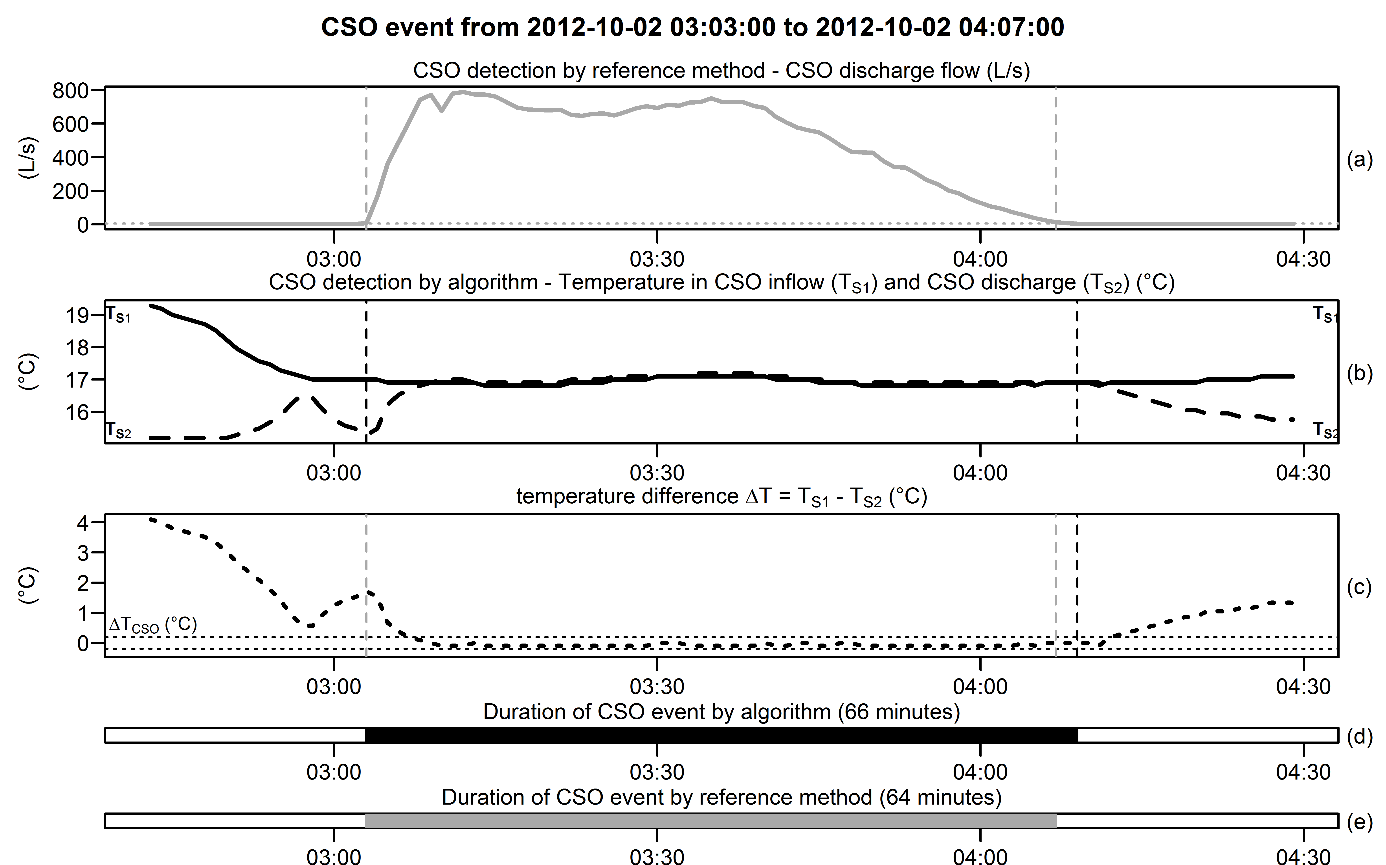


**Fig. A12** Graphical analysis of the detected CSO event #12 (comparison of developed algorithm and reference method) from 2012-10-02 03:03 to 2012-10-02 04:07.


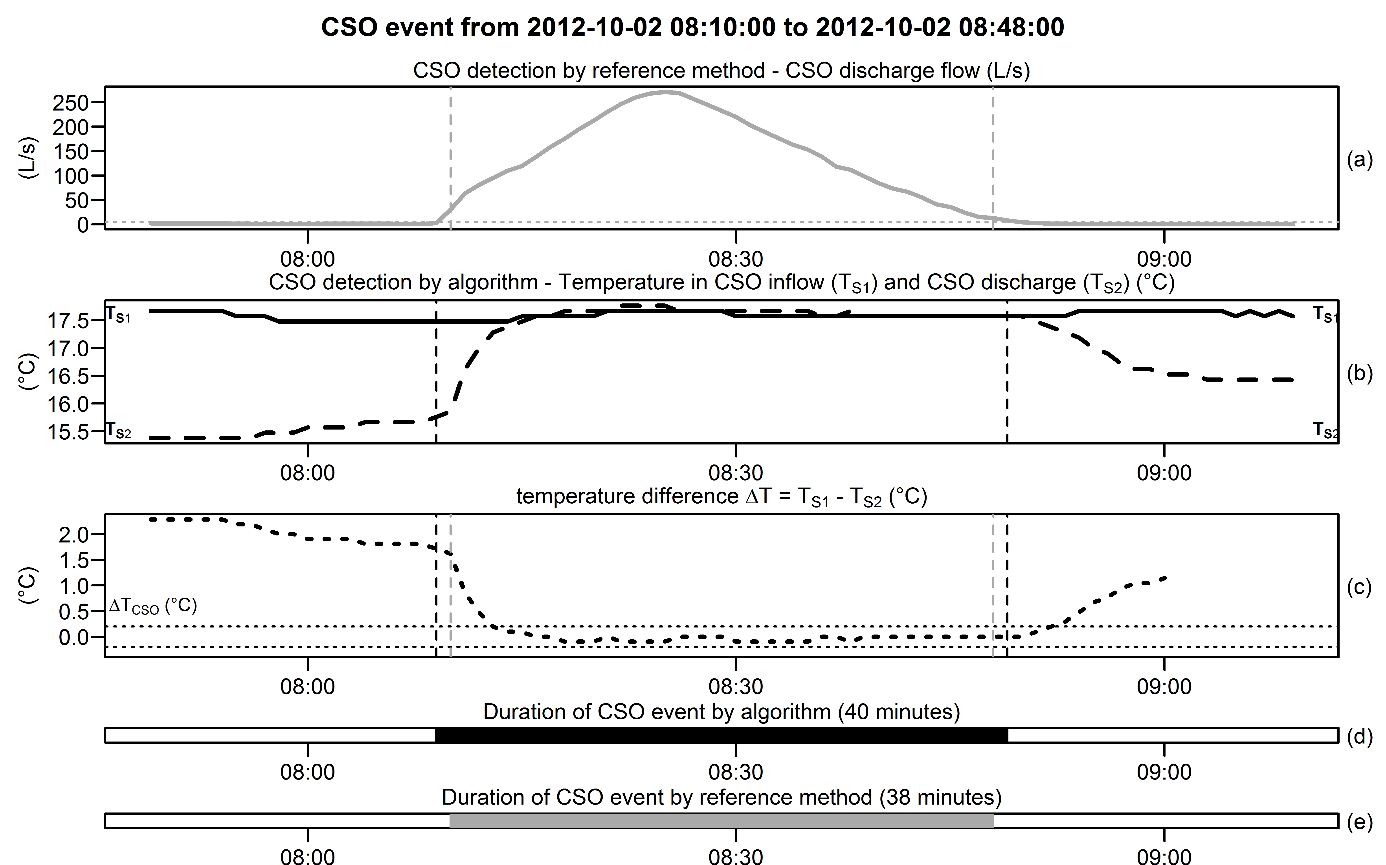


**Fig. A13** Graphical analysis of the detected CSO event #13 (comparison of developed algorithm and reference method) from 2012-10-02 08:10 to 2012-10-02 08:48.


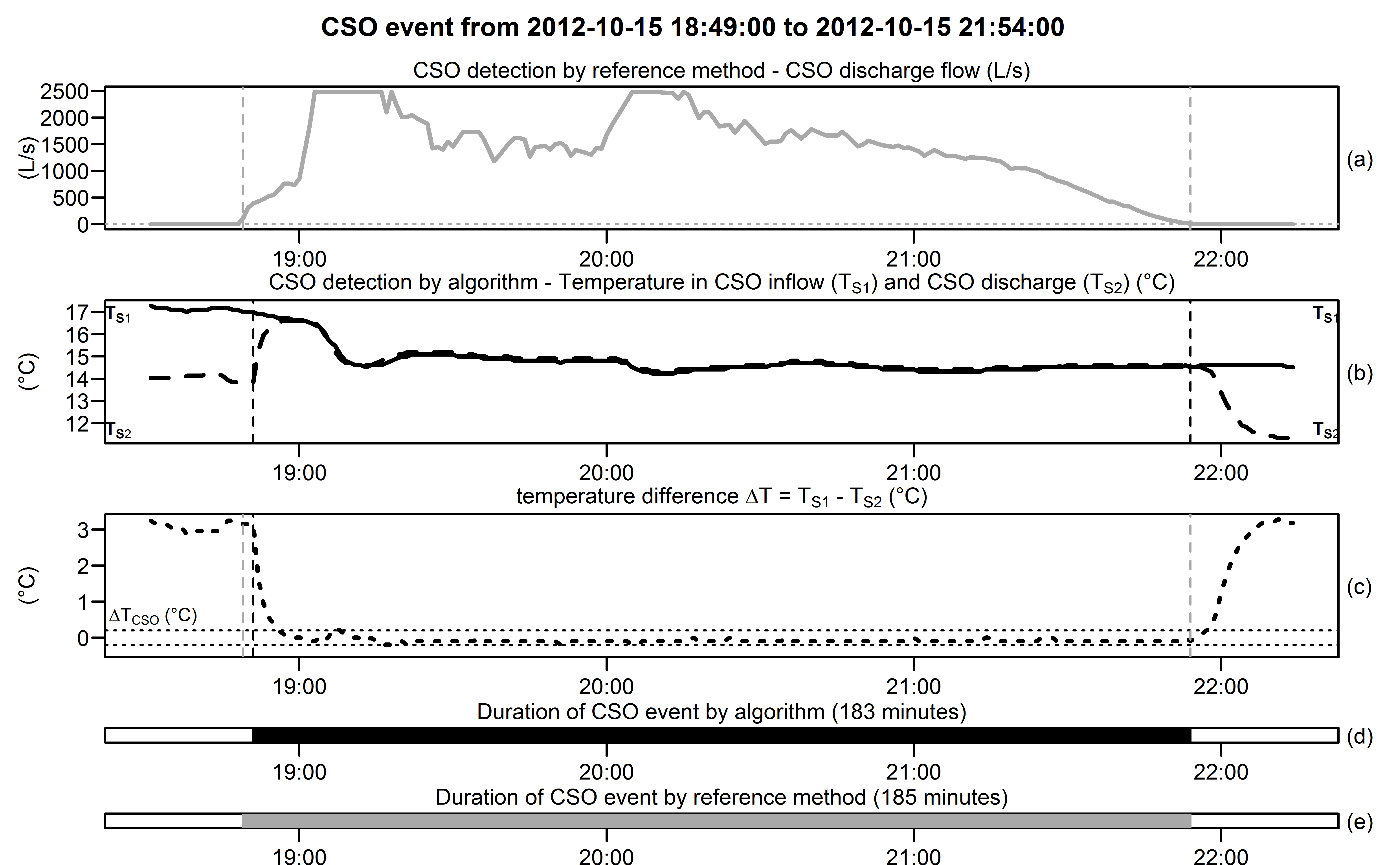


**Fig. A14** Graphical analysis of the detected CSO event #14 (comparison of developed algorithm and reference method) from 2012-10-15 18:49 to 2012-10-15 21:54.


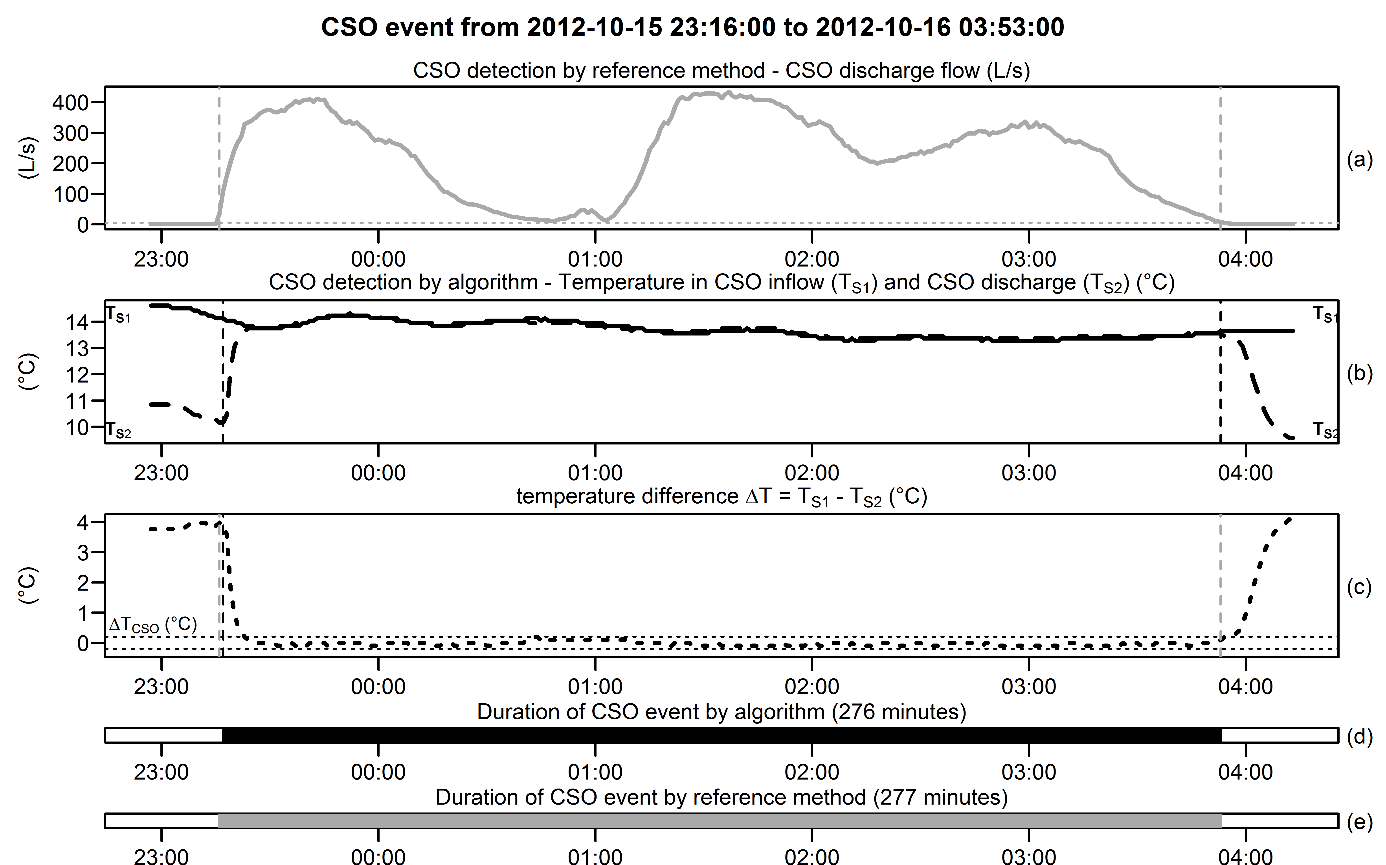


**Fig. A15** Graphical analysis of the detected CSO event #15 (comparison of developed algorithm and reference method) from 2012-10-15 23:16 to 2012-10-16 03:53.


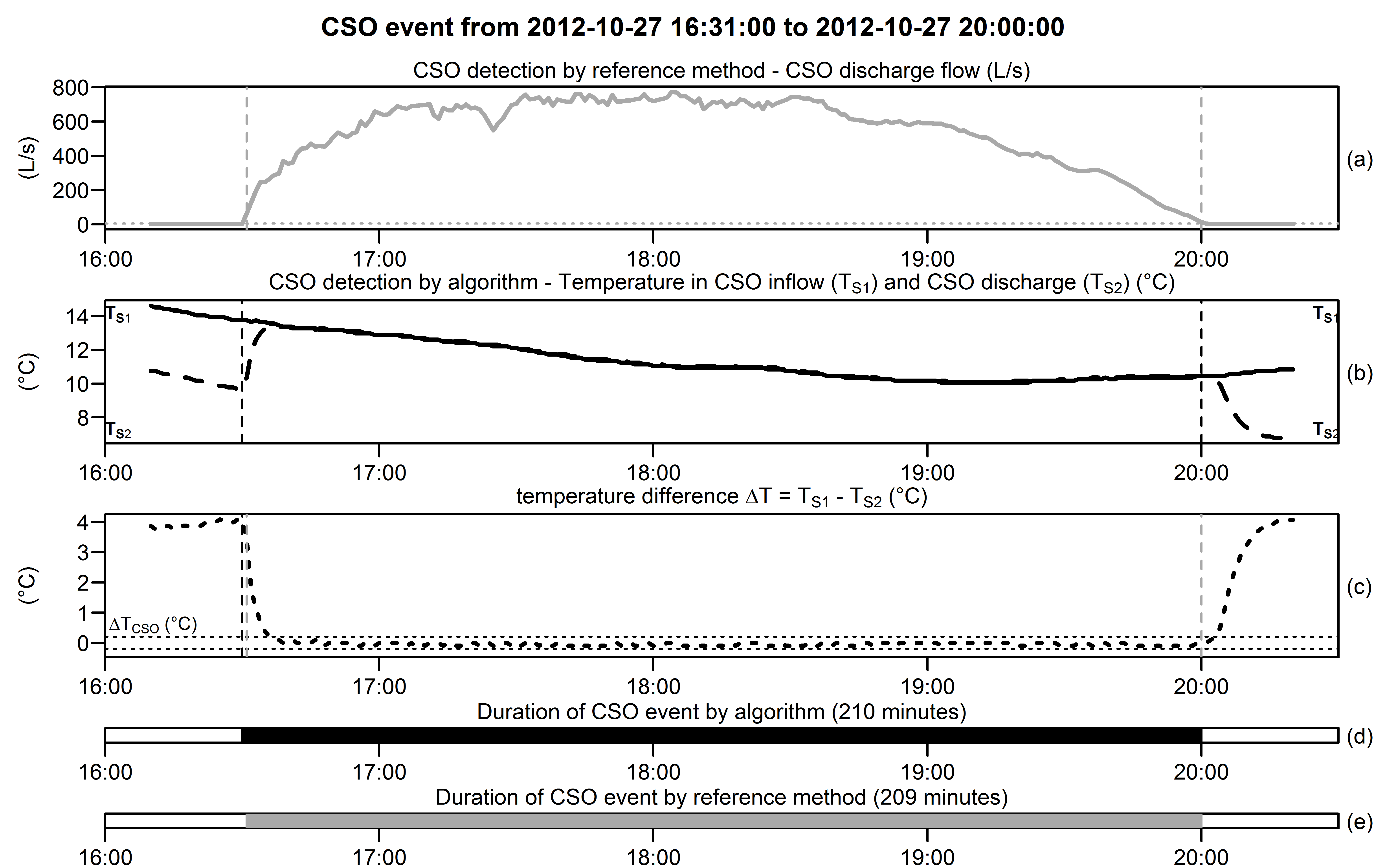


**Fig. A16** Graphical analysis of the detected CSO event #16 (comparison of developed algorithm and reference method) from 2012-10-27 16:31 to 2012-10-27 20:00.


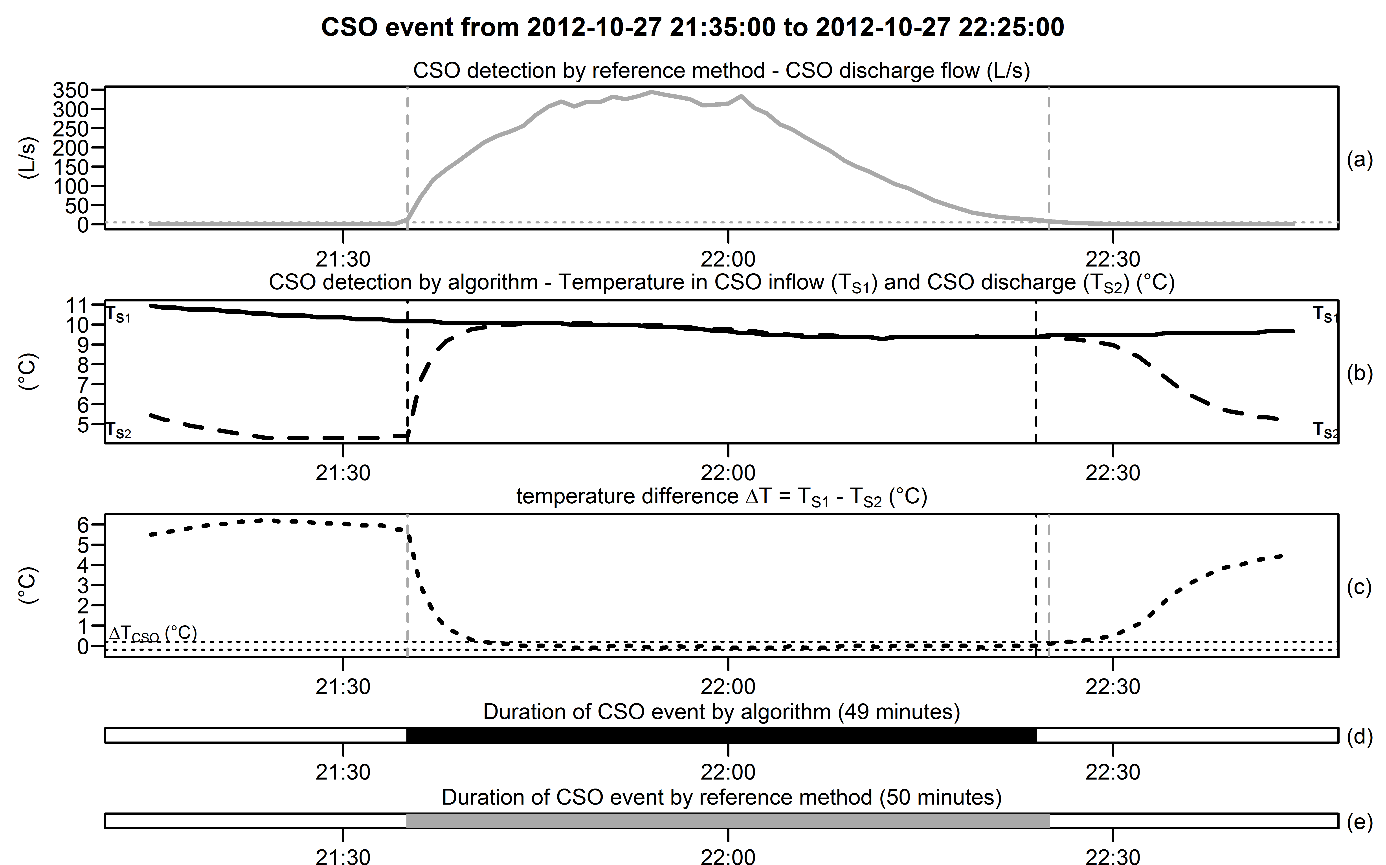


**Fig. A17** Graphical analysis of the detected CSO event #17 (comparison of developed algorithm and reference method) from 2012-10-27 21:35 to 2012-10-27 22:25.


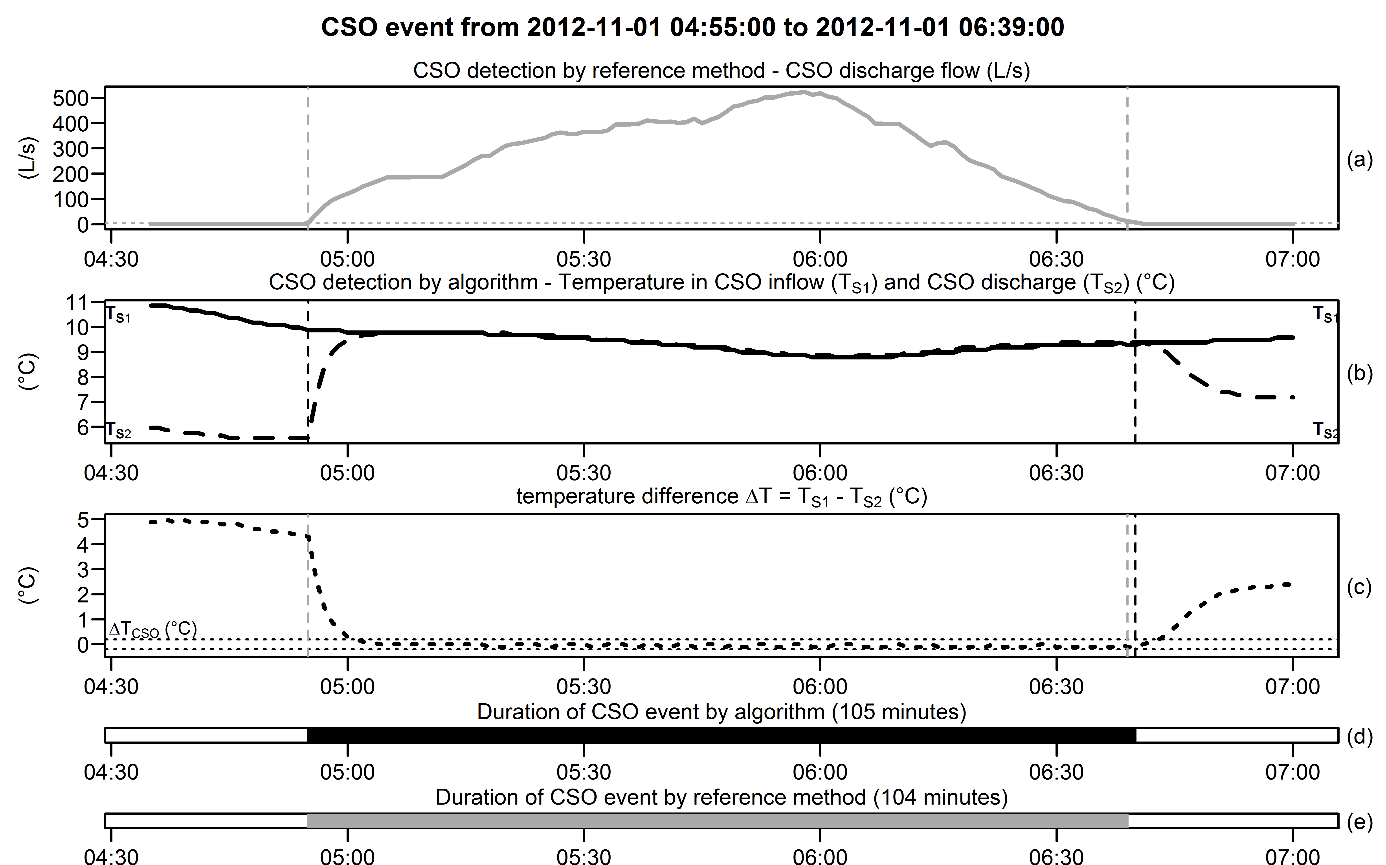


**Fig. A18** Graphical analysis of the detected CSO event #18 (comparison of developed algorithm and reference method) from 2012-11-01 04:55 to 2012-11-01 06:39.


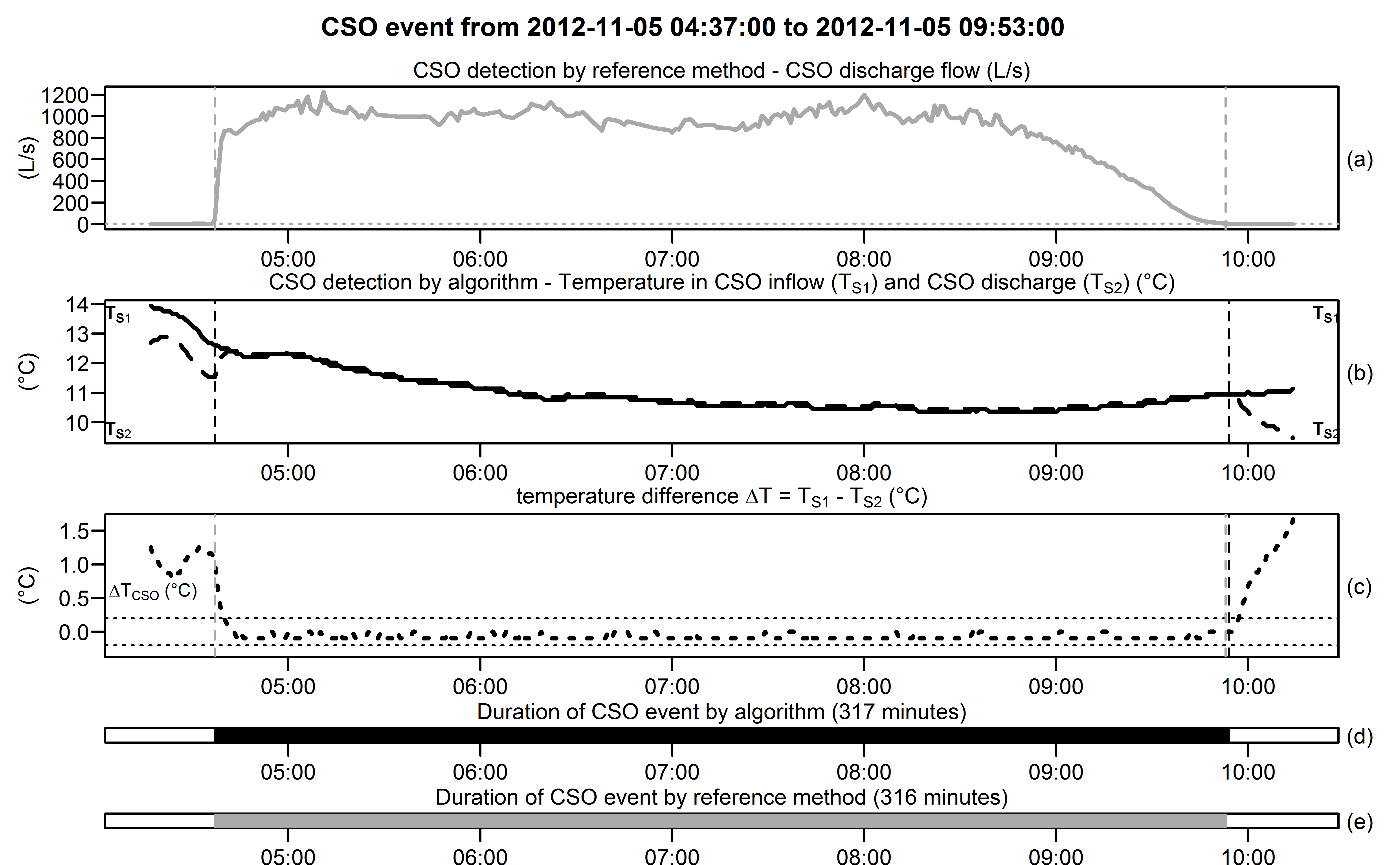


**Fig. A19** Graphical analysis of the detected CSO event #19 (comparison of developed algorithm and reference method) from 2012-11-05 04:37 to 2012-11-05 09:53.


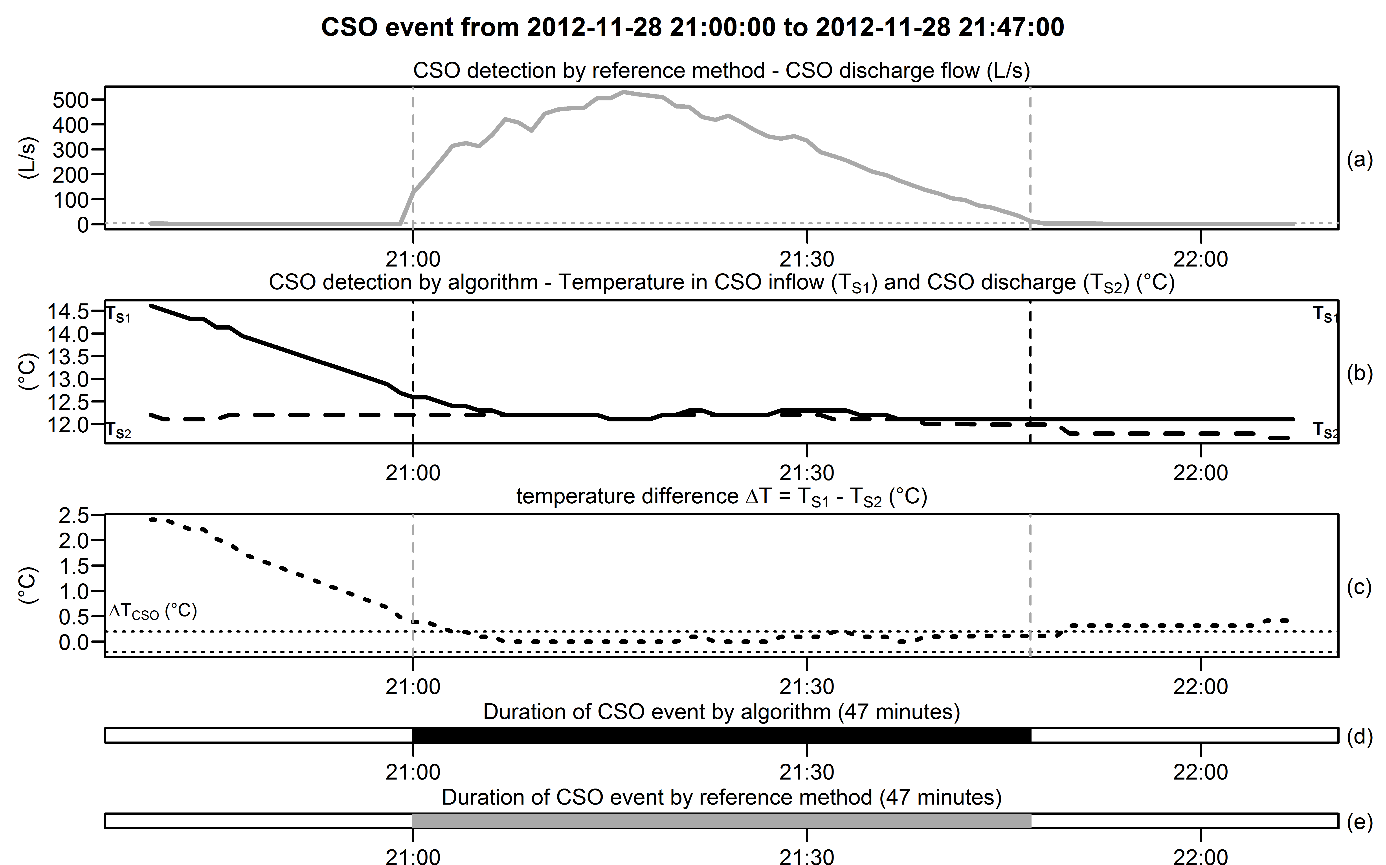


**Fig. A20** Graphical analysis of the detected CSO event #20 (comparison of developed algorithm and reference method) from 2012-11-28 21:00 to 2012-11-28 21:47.
